# Systems profiling reveals recurrently dysregulated cytokine signaling responses in ER+ breast cancer patients’ blood

**DOI:** 10.1038/s41540-024-00447-0

**Published:** 2024-10-10

**Authors:** Brian Orcutt-Jahns, Joao Rodrigues Lima Junior, Emily Lin, Russell C. Rockne, Adina Matache, Sergio Branciamore, Ethan Hung, Andrei S. Rodin, Peter P. Lee, Aaron S. Meyer

**Affiliations:** 1 Department of Bioengineering, University of California, Los Angeles (UCLA), CA USA; 2 Department of Immuno-Oncology, Beckman Research Institute of the City of Hope, Duarte, CA USA; 3 Department of Computational and Quantitative Medicine, Beckman Research Institute of the City of Hope, Duarte, CA USA; 4 Jonsson Comprehensive Cancer Center, Los Angeles (UCLA), CA USA; 5 Eli and Edythe Broad Center of Regenerative Medicine and Stem Cell Research, Los Angeles (UCLA), CA USA

**Keywords:** Cancer, Immunology, Systems analysis

## Abstract

Cytokines operate in concert to maintain immune homeostasis and coordinate immune responses. In cases of ER^+^ breast cancer, peripheral immune cells exhibit altered responses to several cytokines, and these alterations are correlated strongly with patient outcomes. To develop a systems-level understanding of this dysregulation, we measured a panel of cytokine responses and receptor abundances in the peripheral blood of healthy controls and ER^+^ breast cancer patients across immune cell types. Using tensor factorization to model this multidimensional data, we found that breast cancer patients exhibited widespread alterations in response, including drastically reduced response to IL-10 and heightened basal levels of pSmad2/3 and pSTAT4. ER^+^ patients also featured upregulation of PD-L1, IL6Rα, and IL2Rα, among other receptors. Despite this, alterations in response to cytokines were not explained by changes in receptor abundances. Thus, tensor factorization helped to reveal a coordinated reprogramming of the immune system that was consistent across our cohort.

## Introduction

Cytokines are extracellular proteins that mediate cell-to-cell communication within the immune system. Altered levels of various cytokines have been associated with cancer^1^. In addition, we have observed that immune cell cytokine responsiveness is altered in cancer patients, leading to perturbed immune cell function and differentiation^2–5^. In estrogen receptor-positive breast cancer (ER^+^ BC, of any stage) patients, we found that ~40% harbor defects in immune signaling at the time of diagnosis, including altered signaling responses to IL-6, IFNγ, TGFβ, IL-10, and IL-4 in various immune cells^2,6^. Importantly, these peripheral immune defects also reflect the tumor immune microenvironment and predict clinical outcome^7^. The mechanisms that connect altered signaling responses to dysfunctional antitumor immunity remain poorly understood^2,7^. Because cytokines operate in combination in vivo, there is a major gap in our understanding of how the immune system functions from the perspective of cytokine-mediated information flow, and in our ability to predict how alterations might hinder or drive its disruption. A mechanistic understanding of dysfunctional immunity may enable interventions that therapeutically correct these defects, offering a novel approach for improving outcomes in ER^+^ BC patients.

Immune signaling defects in BC patients can be observed through perturbation experiments, wherein patients’ peripheral blood mononuclear cells (PBMCs) are treated with different cytokines, and their signaling responses are measured^2,3,6^. Such experiments can reveal abnormalities in signaling responses which can be reflected in one or several cytokines across multiple cell populations, with heterogeneity across subjects. Thus, while these data are sufficiently rich to uncover defects in immune signaling, the richness itself presents challenges. The multidimensionality of cytokine responses means that any response is dependent on the cytokine itself, cell type, patient, and responsive signaling pathway. Exploring these in combination creates an explosion of variables through their pairwise combination, and typical dimensionality reduction methods (e.g., PCA, t-SNE, U-MAP) have limitations in exploring the data through either their inability to effectively summarize inter-dimensional patterns (PCA) or by sacrificing ease of interpretation due to non-linear properties (t-SNE, U-MAP)^8^.

Dimensionality reduction in tensor form, wherein data is organized into a multi-dimensional array, can preserve the natural organization of profiling experiments that thoroughly characterize these dimensions of cytokine response^8,9^. When data can be arranged in tensor form, tensor decomposition methods can provide improvements in the interpretability of the resulting factors, reduce the data to a greater extent, handle missing values and batch effects, and improve data interpretation^8,10,11^. These properties arise from the ability of tensor decompositions to define the association of each component pattern with each dimension separately^10–13^. By defining component associations with respect to each dimension, tensor decomposition methods are especially effective in integrating datasets of diverse structures with one or more shared dimensions, allowing common patterns to be identified^10,14–18^. Tensor factorization has thus been explored as a natural solution for the analysis of complex, multivariate omics datasets across diverse data modalities including DNA microarray data, transcriptomics, systems serology data, longitudinal ‘omics data, among many others^10,19–21^. As linear methods, tensor decompositions have well-defined properties of convergence, solving, scalability, and interpretation, like matrix-based approaches such as principal component analysis (PCA) or non-negative matrix factorization (NNMF)^8^. However, unlike matrix-based decompositions, tensor decompositions exist in a variety of structural forms, and thus are more flexible in what data formats and contexts they can be used to model^8^.

Here, we profile the cytokine responses and receptor repertoires of immune cells in the peripheral blood from breast cancer patients as compared to healthy controls in a multidimensional manner using canonical polyadic decomposition (CPD). While not commonly applied within the biomedical data sciences, CPD is a tensor decomposition method that can be used to factor n-dimensional, systems-structured data (data gathered consistently across experimental parameters), allowing for the easy visualization of patterns across multiple variables^22^. Applying this method to signaling data allows us to quantify the phosphorylation of signaling products in response to various cytokine treatments concurrently across individual immune cell subsets (“treatments” in this paper will always refer to cytokine stimulation rather than treatment for cancer). Using this approach, we find alterations in cytokine responsiveness across pathways and cell types are widespread in BC compared to healthy subjects, and these changes can be described by signatures across cell types, pathways, and cytokines. Tensor decomposition, specifically CPD, provides a way to define these signatures across these dimensions and allows us to define these changes integratively. We also apply this dimensionality reduction technique to measurements of receptor abundances across immune cell subtypes to identify signatures of altered surface protein abundances across immune populations and patients. With this more complete view, we observe that alterations in the breast cancer cohort’s baseline signaling, receptor amounts, and cytokine responsiveness across cell types exhibit several coordinated features of dysregulated immune signaling. This includes the simultaneous presence of a Th17-like response, or B and CD8^+^ T cells displaying regulatory-like phenotypes.

## Results

### Systematically identifying immune signaling response patterns

**Fig. 1:**
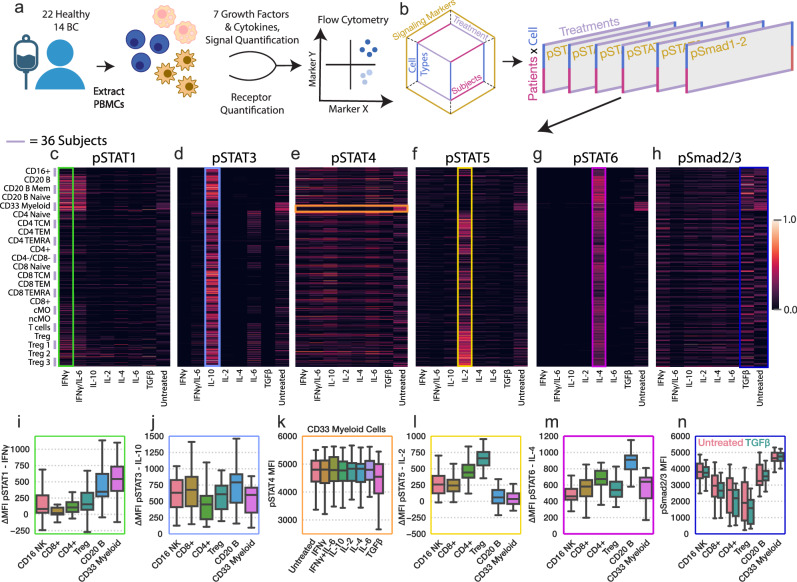
Systematically profiling PBMC cytokine signaling across several dimensions. **a** Schematic of the experimental approach. Human PBMCs were harvested from the healthy and BC cohorts and subsequently treated with a panel of 7 cytokine/growth factor combinations. Response was quantified using 27-channel flow cytometry, through which the response of 23 different cell types was quantified. **b** Schematic of the dataset structure and how it was divided for visualization in (**c**–**f**). Heatmap of phosphorylated STAT1 (**c**), STAT3 (**d**), STAT4 (**e**), STAT5 (**f**), STAT6 (**g**), and Smad2/3 (**h**) measurements for each treatment (X axis), and subject/cell type pair (Y axis). The signal was normalized to the maximum observed signal across subjects. Missing values were imputed for select subjects in response to TGFβ and the IFNγ/IL-6 combination ( ~ 10.4% of total measurements). Missing values are identified in Fig. S2. **i** pSTAT1 responses to IFNγ separated by cell type 9 (n = 36). **j** pSTAT3 responses to IL-10 separated by cell type. **k** pSTAT4 responses to each cytokine in CD33 myeloid cells. **l** pSTAT5 responses to IL-2 separated by cell type. **m** pSTAT6 responses to IL-4 separated by cell type. **n** pSMAD2/3 responses to TGFβ and at baseline, separated by cell type. For all box plots n = 36. For all box plots, the center line denotes the median, the box limits denote the upper and lower quartiles, and the whiskers denote the 1.5x interquartile range.

To systematically characterize the patterns of immune signaling response and the regulatory alterations in ER^+^ breast cancer patients, we analyzed peripheral blood mononuclear cells (PBMCs) from 36 subjects, 22 of which were healthy donors, age 33–71, and 14 of which were newly diagnosed with stage I or II ER^+^ breast cancer, ages 35–71 (Fig. 1a, a full table of patient characteristics can be found in Table S1). We systematically treated the PBMCs with 7 different unique cytokine treatments to profile how the two cohorts differ in their signaling response. The cytokines included IL-2, IL-4, IL-6, IL-10, IFNγ, and TGFβ, as well as a combination of IFNγ and IL-6. These cytokines were selected as they have each been previously shown to have altered signaling activity in BC; however, these studies were conducted using only one cytokine and signaling marker at a time, and included limited staining for cell-type markers, thus limiting their scope^2,3,7,23^. After treatment for 15 min, cells were fixed and stained for 27 different intra- and extracellular markers and then gated into 23 distinct populations (Fig. S1). Alongside various cell type markers, the phosphorylation responses of key transcription factors downstream of our cytokines were quantified (pSTAT1, pSTAT3, pSTAT4, pSTAT5, pSTAT6, and pSmad2/3).

Using these data, we first confirmed that several canonical signaling trends were present in data collected from both healthy and BC cohorts. We isolated subsets of our data and collapsed it into matrices to visualize how cell types from different subjects (Y axis) responded to each cytokine treatment (X axis) (Fig. 1b–h). For instance, STAT1 phosphorylation was especially responsive to IFNγ stimulation in B cells and CD33-positive myeloid cells (Fig. 1c, i, green box). As expected, IL-10 stimulation resulted in STAT3 phosphorylation that was most abundant in B and CD8^+^ cells (Fig. 1d, j, blue box)^24^. All treatments here resulted in minimal STAT4 phosphorylation; no statistically significant responses were measured even in CD33 myeloid cells, the population which generally responded most strongly in STAT4 response, a finding which is unsurprising, as STAT4 phosphorylation is most strongly induced by two cytokines which we did not profile, IL-12 and IFNα (Fig. 1e, k, orange box)^25^. IL-2 induced STAT5 phosphorylation and was found to occur with the greatest magnitude in regulatory T cells (Tregs), which are known to be sensitive to IL-2 stimulation (Fig. 1f, l, yellow box). This pattern was consistent among T_reg_ subsets I, II, and III, each of which have been shown to play distinct roles in immunosuppression and correlate in distinct manners with breast cancer outcome^7,26^. STAT6 was found to be most strongly phosphorylated by IL-4; however, there was substantial variation in response within CD8^+^ cells across subjects (Fig. 1g, m purple box). Finally, TGFβ elicited minimal Smad2/3 phosphorylation across all cell types (Fig. 1h, n, dark blue box). However, baseline measurement of both STAT4 and SMAD2/3 phosphorylation proved to be consistently variable across populations, suggesting that our experimental approach captured these markers effectively. Although collapsing the data into matrices allowed us to visualize the most large-scale patterns, an analysis technique which accounts for the systems-structured data is required to identify the shared and distinguishable response patterns between the healthy and BC cohorts across all the parameters measured (cell types, treatments, subjects).

### CPD reveals coordinated and substantial BC-associated patterns of signaling response

**Fig. 2:**
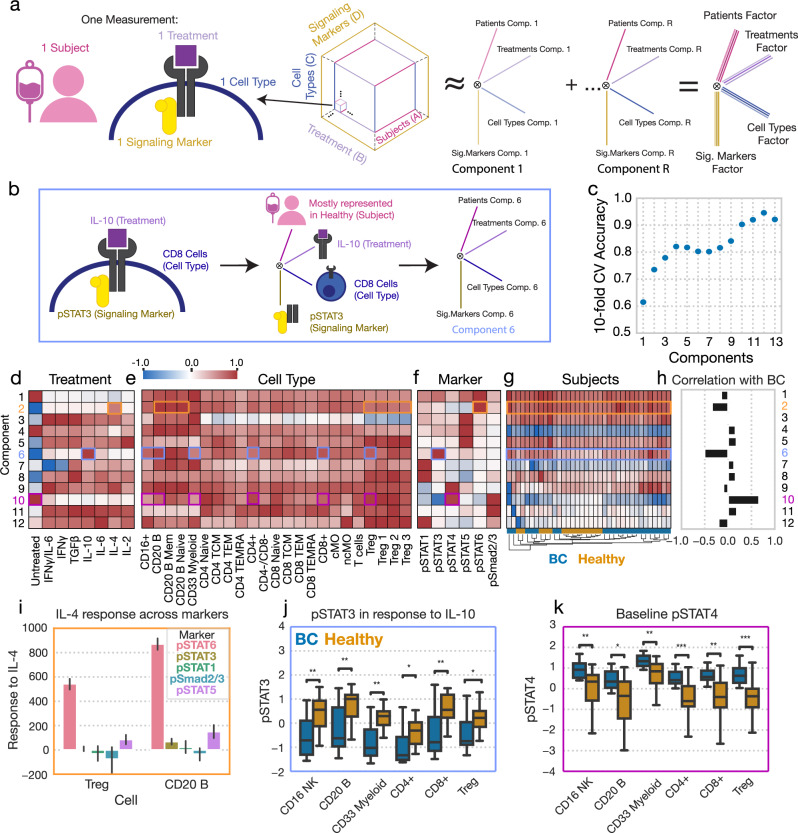
Canonical polyadic decomposition (CPD) of cytokine response identifies several patterns of cytokine response strongly associated with BC. **a** Schematic of the CPD. The signaling data is organized into a four-dimensional tensor with axes for each subject, treatment, cell type, and signaling marker. This tensor is reduced into the sum of the outer products of vectors (components) associated with each dimension. **b** Schematic demonstrating the interpretation of a single component, component 6. **c** The accuracy of a logistic regression classifier upon tenfold cross-validation, using the CPD subject factors with varying numbers of components. (**d**-**f**) Component values for each treatment (**d**), cell type (**e**), and signaling marker (**f**). **g** A heatmap of the subject factor matrix, with the subjects hierarchically clustered. Subject status is indicated by the coloring along the bottom. **h** The univariate correlation of each subject component with BC status (healthy = 0, BC = 1). **i** Subject responses of each signaling marker to IL-4 in Tregs and B cells. Error bars in this figure represent standard deviation (n = 36). **j** pSTAT3 responses to IL-10 across cell types, separated by subject disease status (healthy n = 22, BC n = 14). **k** Baseline pSTAT4 across cell types, separated by subject disease status (healthy n = 22, BC n = 14). Components and their associated plots are denoted by their color. For all box plots, the center line denotes the median, the box limits denote the upper and lower quartiles, and the whiskers denote the 1.5x interquartile range. Significance was derived using the Mann–Whitney U test comparing those measurements from healthy donors to those of BC patients. *, **, and *** represent p values less than 0.05, 0.005, and 0.0005, respectively.

Examining individual cytokine responses or responsive pathways can only provide a limited picture of how responses vary, which is a common challenge with profiling data collected across several experimental parameters^27^. Therefore, we sought to identify patterns of response variation using an approach that explicitly accounts for data gathered across multiple experimental conditions. To visualize the variation in responses across each subject, cytokine treatment, cell type, and signaling pathway, we utilized CPD. CPD factors an n-dimensional tensor, in this case four-dimensional, into the sum of vector outer products (Fig. 2a). Analogous to matrix-based methods like PCA, each set of vectors represents a pattern/component, and individual vectors each associate with a specific dimension, describing how the pattern is represented along the specified axis. As a result, each component encodes a distinct response pattern across dimensions (Fig. 2b).

While the factors for each dimension coordinately explain the data, the subject factors specifically describe how these patterns vary between the healthy and BC cohorts. As the number of components is a tunable setting within CPD to explain more or less of the variation, we chose a decomposition rank that optimized the prediction accuracy of disease status (Fig. 2c). To classify subjects, a logistic regression model was fit to predict disease status by using subject factors as input features. Using this approach, we found that a decomposition of rank 12 was optimal, with 94% accuracy on tenfold cross-validation (Fig. 2c). Further, about 80% of the variance in our original dataset could be captured with 12 components (Fig. S3). Additionally, CPD led to a more concise representation of the data compared to PCA, explaining a similar amount of the data compared to PCA at less than 1% of the size (Fig. S3a, b). We also explored whether Tucker decomposition would efficiently reduce the size of our data, and found that predictably, such decomposition was able to more efficiently explain the variance in our response data (Fig, S3c). However, when we examined the accuracy with which Tucker-derived patient factors could predict BC status, we found that such factors were similarly informative to those derived through CPD—thus, given that CPD is much more interpretable than Tucker factorization, we proceeded with our CPD-based analysis^8,9^. We also confirmed the appropriateness of CPD as a representation of the data as well as that the size of the factorization is appropriate, as CPD was shown to accurately impute missing data up to and past 12 components. This indicated that the CPD was able to recover and recapitulate missing data effectively, and thus that there were no superfluous or redundant components at such decomposition ranks (Fig. S4a, b). Furthermore, we confirmed that our rank 12 decomposition led to consistent factors by resampling our data via jackknife sampling across patients, performing CPD on those subsamples, and calculating the factor match score comparing our full decomposition to subsampled decompositions. Here, we observed agreement between the full and subsample decompositions (mean FMS = 0.74, SD = 0.09), confirming the stability of our rank 12 decomposition.

As signaling responses can also be measured as the fold change in signaling product rather than the total signal (used above), we tested a similar modeling approach using responses recorded as fold changes, and found that the resulting model was similarly, but slightly less, predictive (Fig. S5). Interestingly, when using a similar approach with cell type abundances as inputs, we were unable to identify patterns associated with BC status, suggesting that no significant differences in the composition of cell types exist between the healthy and BC cohorts (Fig. S6).

The CPD factors effectively mapped expected signaling trends to specific subjects, treatments, cell types, and signaling markers (Fig. 2d–h). Having performed hierarchical clustering on the subject factors, we found that subjects largely grouped according to disease status (Fig. 2g). One can readily interpret the resulting factor plots by tracing an individual component across each dimension. For instance, component 2 (orange boxes) describes variation with respect to IL-4 treatment (Fig. 2d) and STAT6 phosphorylation (Fig. 2f). This pattern was present in all cell types, though more so in B cells, compared to other cell types where the pattern was less prominent, such as in Tregs; this finding was reflected in the raw data (Fig. 2e, i). It was also consistent across all subjects in both BC and healthy cohorts (Fig. 2g).

We next wondered whether the patterns described by our components varied according to disease status. By examining the univariate correlation with BC status (Fig. 2h), we found that component 6 (blue boxes), which represented STAT3 phosphorylation (Fig. 2f) in response to IL-10 (Fig. 2d) across many cell types (Fig. 2e), was among the components which separated BC patients from healthy donors most effectively, a difference reflected in our raw data (Fig. 2h, j). Using this approach, we also found that component 10 (purple boxes), which is associated with baseline abundances of pSTAT4 and pSmad2/3 also largely separated BC patients from healthy donors (Fig. 2h). This association with disease was reflected in the raw data when stratifying basal pSTAT4 levels by subject group (Fig. 2k). It is important to note that while the dysregulation described by components 6 and 10 are both correlated with disease status and are represented similarly across our broadest categories of cell types (Fig. 2j/k), their association with specific immune subtypes (i.e., CD4^+^ subtypes) was distinct.

Taken together, CPD effectively reduced the response data into coordinated patterns of cytokine response, which in turn associated strongly with BC status. CPD identified that BC patients are most prominently distinguished by their reduced IL-10 response across many cell populations, particularly in B and CD8^+^ cells, (component 6) (Fig. 2i) and basal pSmad2/3 and pSTAT4 across many populations (component 10) (Fig. 2k). These alterations are reflected individually within a full table of features which we found to be associated with BC status in univariate fashion in Table 1, as well as shown in Fig. S8. However, it is important to note that our CPD results indicate that these features better define and separate healthy from BC patients when defined as combinations of variables/components; such components which we specifically highlight in the text are shown in Table 2.

**Table 1:** Full table of variables which distinguish breast cancer patients from healthy in univariate manner

Pattern	Cell types
Decreased pSTAT3 response to IL-10	Pan-cell type, and particularly in CD8^+^ cells
Increased basal pSTAT4	Pan-cell type, and particularly in CD8^+^ cells
Increased basal pSmad2/3	Pan-cell type, and particularly in CD8^+^ cells
Increased PD-L1 abundance	B and CD8^+^ effector cells
Increased IL2Rα abundance	Treg cells, CD4^+^ cells
Increased IL6Rα abundance	CD16^+^, B, CD8^+^ cells, and monocytes
Increased IL2Rβ abundance	B cells and CD8^+^ cells
Decreased IFNγR1 abundance	CD16^+^ cells, CD4^+^ cells

**Table 2:** Full table of highlighted components associated with breast cancer

Component	Cell types	Cytokines	Signaling Marker/Receptor	Figure
Signaling Component 5	T cells, and Tregs	IL-2	pSTAT5	2, 3
Signaling Component 6	Pan-cell type, and particularly in CD8^+^ and CD20 B cells	IL-10, Basal	pSTAT3	2, 3
Signaling Component 10	Pan-cell type, and particularly in CD8^+^ and CD20 B cells	Basal	pSTAT3, pSTAT6, pSmad2/3	2, 3
Receptor Component 2	CD20 B cells, CD8^+^ cells, Tregs	N/A	PD-L1, IL6Rα, IL2Rβ	4, 5

### CPD reveals complex correlations and relationships both between and within pathways dysregulated in breast cancer

**Fig. 3:**
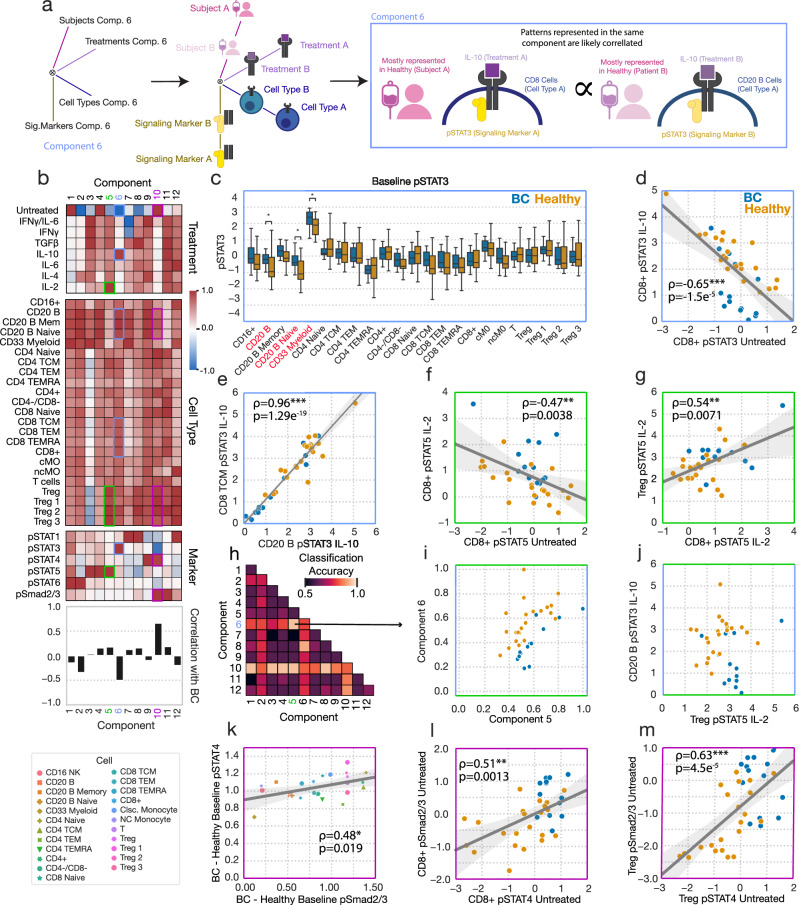
Alterations in immune signaling responsiveness in BC are more prominently reflected in coordinated patterns of signaling response changes. **a** Schematic of showing how CPD factors can be used to discover coordinated patterns of response across data dimensions. **b** Cytokine response data factorization from Fig. 2. **c** Baseline pSTAT3 in untreated cells across cell types, separated by subject status. For this box plot, the center line denotes the median, the box limits denote the upper and lower quartiles, and the whiskers denote the 1.5x interquartile range. Significance was derived using the Mann–Whitney U test comparing those measurements from healthy donors to those of BC patients (healthy n = 22, BC n = 14). **d** Baseline pSTAT3 versus IL-10-induced pSTAT3 in CD8^+^ cells for each subject (n = 36). **e** pSTAT3 response to IL-10 in B cells versus CD8 TCM for each subject. **f** Baseline untreated versus IL-2-induced STAT5 phosphorylation in CD8^+^ cells for each subject. **g** IL-2-induced pSTAT5 in CD8^+^ cells versus Tregs for each subject. **h** Classification accuracy (tenfold CV) for logistic regression classifiers using all pairs of subject components. **i** Components 5 versus 6 across subjects from the CPD factorization in Fig. 2. **j** IL-2-induced pSTAT5 in Tregs versus IL-10-induced pSTAT3 in B cells. **k** The difference in average Smad2/3 phosphorylation between the BC and healthy cohorts versus the same quantity for STAT4 phosphorylation, plotted for each cell type (n = 23). (**l**, **m**) Baseline pSmad2/3 versus pSTAT4 across subjects in CD8^+^ (**l**) and Tregs (**m**) (n = 36). All reported correlations are Pearson correlations. Components and their associated plots are denoted by their color. *, **, and *** represent p values less than 0.05, 0.005, and 0.0005, respectively.

After finding that the subject-associated factors were overall predictive of disease status, we sought to further dissect the specific changes associated with BC using the CPD factorization as a guide (Fig. 3a, b). While we could observe the prominent difference between BC patients and healthy donors in IL-10-induced pSTAT3 in a per-measurement analysis without the aid of CPD (Fig. 2i), we wondered whether the CPD results offer additional insight into this prominent difference. First, we noted that the component associated with this reduction in response (component 6, blue boxes) was also associated with an increase in baseline pSTAT3 (Fig. 3b). Despite this, only a few cell types featured a statistically significant difference in baseline pSTAT3 associated with BC (Fig. 3c). However, comparing the baseline versus induced pSTAT3 in CD8^+^ cells, we indeed observed that, across subjects, increased baseline pSTAT3 was associated with reduced response to IL-10 (Fig. 3d). Second, each component may represent multiple coordinated patterns that are shared across cell types and subjects, allowing for the identification of correlation between complex, multivariate patterns of response. To examine this, we selected two individual patterns implicated within component 6 (IL-10 response in B cells and CD8 TCMs) and plotted the correlation between them across subjects. We observed that cell types were strongly correlated in their response magnitude (Fig. 3e). This inter-population correlation was also observed to be consistently strong between other populations implicated in component 6 (CD8^+^ cells and subsets thereof, B cells and subsets thereof). Therefore, CPD provided the added insight that reduced IL-10 response is associated with an increase in the baseline level of pSTAT3 and is coordinated across cell types, varying in magnitude across subjects according to their disease status.

The results of CPD also suggest that patterns of altered signaling in combination could better distinguish healthy donors from BC patients than any single pattern. For instance, when looking at component 5 (green boxes), IL-2 response was increased within CD8^+^ cells in BC patients. We found that using baseline versus IL-2-induced pSTAT5 (Fig. 3f), or CD8^+^ versus Treg responses (Fig. 3g), incompletely separated BC patients from healthy donors. However, BC prediction accuracy was much enhanced when component 5 and component 6 were considered in combination (Fig. 3h). Component 5 (associated with IL-2-induced pSTAT5, particularly in Tregs) led to almost perfect separation when combined with component 6 (associated with IL-10-induced pSTAT3 particularly in CD8^+^ and CD20 B cells) (Fig. 3i, j). Through this, we observed that CPD, though unsupervised, can better define subject groups by defining integrative signatures of response.

Furthermore, we found that CPD-defined factors could additionally identify coordinated changes across subjects, cell types, and pathways. Component 10 (purple boxes) represents an increase in basal Smad2/3 and STAT4 phosphorylation in untreated cells across most cell types (Fig. 3b). While a per-measurement analysis reveals these same differences, the CPD results reveal that these alterations are coordinated in the magnitude of the difference not only across cell types (Fig. 3k, S8) but also across subjects (Fig. 3l, m). Therefore, profiling and analyzing these differences multidimensionally reveals how signatures of alteration in signaling responses are coordinated across dimensions and improves our ability to resolve these differences.

While single components that are highlighted in the text are described in Table 2, the above analysis shows that even multidimensional components better define alterations in immune signaling when considered in combination. Wielding these multivariate components in combination both allows one to more effectively define the differences between the healthy and disease cohorts and biologically interpret the basis for those connected patterns.

### CPD identifies coordinated patterns of PBMC receptor abundance variation

Cell surface receptor abundances vary broadly across cell types and define which cells are capable of cytokine and growth factor responses^28^. Therefore, we next investigated whether receptor abundance itself may associate with cancer status or define the differences in cytokine response. For instance, reduced IL-6 signaling in BC patient PBMCs, associated with poor clinical prognosis, has correlated with reduced IL-6 receptor abundance^2^. To investigate whether similar differences were present across our subject samples, we measured receptor abundance profiles in each subject and cell type across the cohort. Cells were stained for the cognate receptors of each cytokine stimulant (e.g., IL10R, TGFβRII), receptors with signaling pathways converging on our cohort of measured transcription factors (e.g., IL12RII–pSTAT4, IL7Ra–pSTAT3/5), as well as the checkpoint proteins PD-1 and PD-L1.

**Fig. 4:**
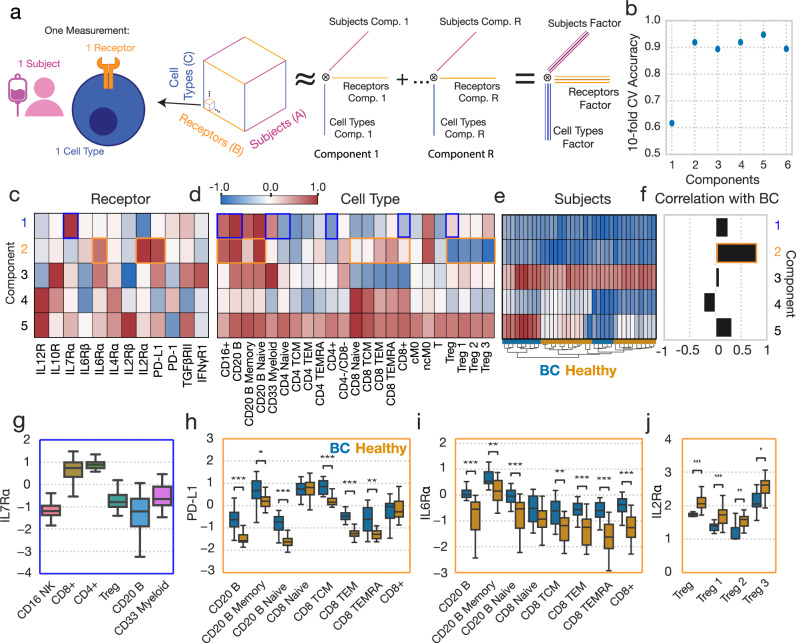
CPD reveals patterns of receptor abundance variation. **a** Schematic of CPD. Receptor data is organized into a three-dimensional tensor with axes of subject, cell type, and receptor. This tensor is reduced into sum of the outer products of rank 1 tensors (components), allowing for easy visualization. **b** The accuracy of a logistic regression classifier upon tenfold cross-validation, using the CPD subject factors with varying numbers of components. (**c**, **d**) Component values for each receptor (**c**) and cell type (**d**). **e** A heatmap of the subject factor matrix, with the subjects hierarchically clustered. Subject status is indicated by the coloring along the bottom. **f** The univariate correlations of each component with subject disease status (healthy = 0, BC = 1). **g** IL7Rα response across cell types (n = 36). **h** PD-L1 abundance across cell types, separated by subject disease status (healthy n = 22, BC n = 14). **i** IL6Rα abundance across cell types, separated by subject disease status. **j** IL2Rα abundance across cell types, separated by subject disease status. For all box plots, the center line denotes the median, the box limits denote the upper and lower quartiles, and the whiskers denote the 1.5x interquartile range. Significance was derived using the Mann–Whitney U test comparing those measurements from healthy donors to those of BC patients. Components and their associated plots are denoted by their color. *, **, and *** represent p values less than 0.05, 0.005, and 0.0005, respectively.

These data can be naturally organized into a three-dimensional tensor, with subject, cell type, and receptor dimensions (Fig. 4a). Five components explained over 60% of the variance in our dataset (Fig. S7a). As with the cytokine response data, tensor factorization reduced the dataset more efficiently than PCA (Fig. S7b). We again validated our CPD approach for the receptor dataset by testing the model’s ability to impute missing values (Fig. S4c, d). The five-component model was selected based on its optimal association with BC disease as well as for its more complete summarization of the receptor abundance datasets than smaller and slightly less predictive factorizations (Fig. 4b, Fig. S7). We summarized and visualized the receptor abundance data using a tensor decomposition of rank 5 (Fig. 4c–f). As before, we also compared the efficiency of Tucker factorization of our receptor dataset to that of CPD, and the accuracy with which the subject-factors could be used to classify BC patients, and found that despite increased decomposition efficiency, Tucker derived factors were equivalently predictive to CPD-derived factors (Fig. S7c, d). Again, we elected to proceed with our CPD-based analysis due to the relative ease with which CPD results can be interpreted. We calculated the FMS of jackknife subsampling for our receptor tensor across patients, and observed strong consistency in our resulting factors (mean FMS = 0.93, SD = 0.12).

Although the five-component model produces components that are highly associated with BC disease, hierarchical clustering of the subject factors broke both the healthy donors and cancer patients into two groups each, reflecting that subject-to-subject variation was present beyond just that contained in the highly BC-specific component 2 (orange boxes) (Fig. 4e). Univariate correlations with BC status identified that component 2 almost distinguishes perfectly the BC and healthy subjects, where subjects assigned larger component 2 values are more likely to be members of the BC cohort (Fig. 4f). Component 5, which described IL12R and IL2Rβ abundances, particularly in CD8^+^ cells, was also identified to have a unique relationship with BC status, in that BC patients featured either very positive or very negative component 5 scores (Fig. 4f).

As before, one can trace a single component across the factor matrices to make inferences about that pattern within the dataset. For instance, component 1 (blue boxes) represents variation in IL7Rα across cell types, and identifies previously reported patterns of abundance, such as increased abundances in CD4^+^ cells^11^ (Fig. 4g). Component 2, the strongest predictor of disease status, identified a litany of patterns found to vary according to disease status; namely, it identified variation in PD-L1, IL6Rα, and IL2Rα primarily in CD4^+^, CD8^+^, Treg, and B cell populations (Fig. 4c). In BC patients, PD-L1 was elevated in TCM, TEM, and TEMRA-positive CD8 cells, though not in the naïve subset, and was upregulated in several B cell subsets (Fig. 4h). IL6Rα was also found in higher amounts in roughly the same cell subsets (Fig. 4i). IL2Rɑ, by contrast, decreased with BC in regulatory T cell subsets (Fig. 4j), consistent with the cell populations’ negative weighting on component 2 (Fig. 4d). We observed that variation in several receptors was summarized by single components, such as the three receptors associated with component 2, indicating correlations in these receptors’ expression across subjects and cell types (Fig. 4c, h–j). Overall, CPD succinctly summarized these patterns of receptor variation across both cell types and subjects, and again identified patterns which varied according to subject BC status. As with our findings with respect to alterations associated with BC found in cytokine responses, alterations in receptor abundances which distinguish healthy donors from diseased patients in a univariate manner are compiled in Tables 1, 2 highlights multivariate receptor components associated with BC.

### Dissection of CPD factorization reveals concerted immunologic reprogramming

**Fig. 5:**
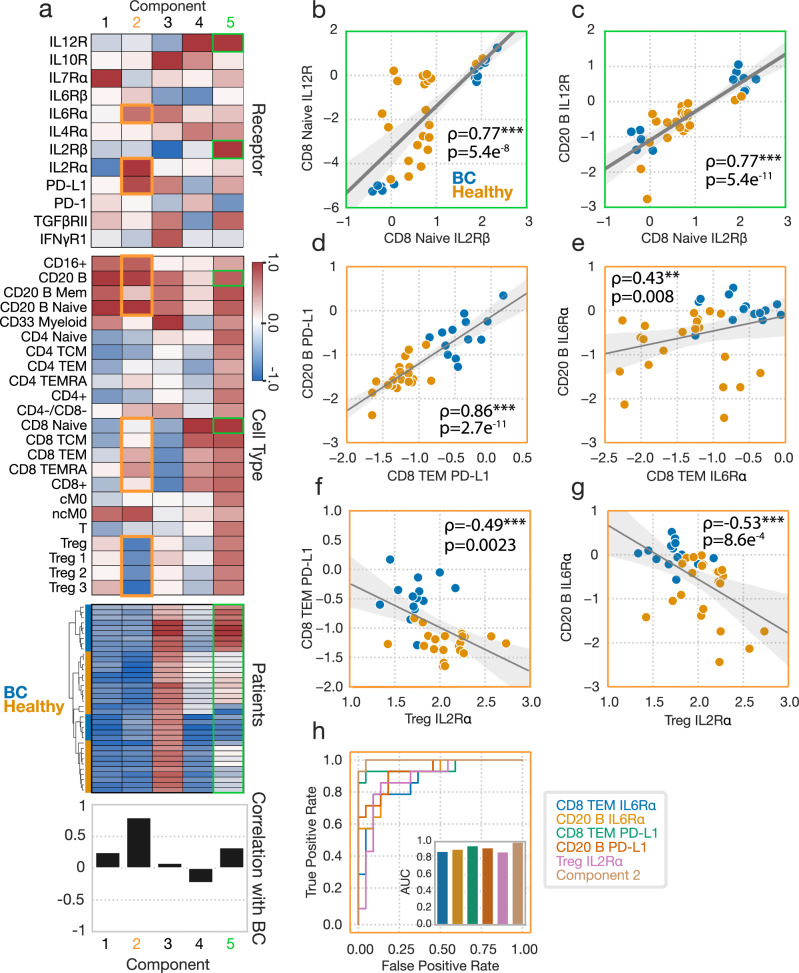
Dissecting the patterns of receptor variation define concerted molecular programs. **a** Receptor abundance data factorization from Fig. 4. **b** IL2Rβ versus IL12R abundances in CD8 naïve cells across subjects (n = 36). All reported correlations are Pearson correlations. **c** IL2Rβ abundances in CD8 naïve cells versus IL12R abundance in B cells for each subject. (**b**-**e**) Correlation across subjects between CD8 naïve IL2Rβ and IL12R (**b**), CD8 naïve IL6Rα and B cell IL6Ra (**c**), CD8 TEM PD-L1 and B cell PD-L1 (**d**), CD8 IL6Rα and CD20 B IL6Rα (**e**), Treg IL2Rɑ and CD8 TEM PD-L1 (**f**), and Treg IL2Rɑ and CD20 B IL6Rα (**g**). **h** ROC curve for the separation provided by several of the receptor amounts measurements, alongside component 2. AUC plots for each feature are also shown. Components and their associated plots are denoted by their color. *, **, and *** represent p values less than 0.05, 0.005, and 0.0005, respectively.

Using the CPD results as a guide (Fig. 5a), we further dissected the patterns of receptor variation. Given that IL2Rɑ was represented by the same BC-associated component as IL6Rα and PD-L1 in the CPD analysis, but was dysregulated in populations distinct from those in which IL6Rα and PD-L1 were dysregulated in univariate analyses (Fig. 4h–j), we wondered whether a per-measurement view masked additional relationships among these measurements. Each component in CPD represents variation shared among subjects, receptors, and cell types. Therefore, we explored whether we could observe these coordinated changes within the raw receptor data to make insights regarding programs of receptor expression. For example, as suggested by component 5 (green boxes), we found a strong correlation between IL12R and IL2Rβ abundances in naïve CD8 cells (Fig. 5b), as well as between IL12R abundance in CD20 cells and IL2Rβ abundance in naïve CD8 cells (Fig. 5c). Furthermore, we observed a distinct bimodal separation among BC patients across each of these correlated receptor abundances (Fig. 5b, c). This bimodal separation can also be seen when looking at how the hierarchical clustering of the subjects factor broke the subject conditions up into two groups each (Fig. 5a). This clustering did not appear to have any relationship with patient age, and matched patient PR status was unavailable, leaving the origin of this bimodal relationship unclear. This observation again demonstrated the utility that CPD has in identifying relationships between protein abundances across immune populations which univariate analysis alone may fail to identify.

We next sought to further break down component 2 (orange boxes), which has the highest univariate correlation with BC status. Comparing abundances of a single surface protein across populations, we observed that PD-L1 abundance was strongly correlated across subjects between CD8 TEM and B cells (Fig. 5d), as was, albeit to a lesser degree, IL6Rα abundance (Fig. 5e). Furthermore, the pattern of coordinated changes extended to comparisons across both receptors and cell types. We observed correlations across subjects when comparing the abundance of IL2Rɑ among Tregs against PD-L1 in CD8 TEM cells (Fig. 5f). As another example, we observed a negative correlation between IL2Rɑ abundance in Tregs and IL6Rɑ abundance in B cells (Fig. 5g). These results indicate that the CPD-defined components indeed represent coordinated changes within and across cytokine pathways and immune populations.

Lastly, we surmised that the coordinated changes across receptors and cell types might better distinguish BC patients from healthy donors either due to averaging over measurement noise or capturing inherently multivariate patterns. Indeed, when comparing the classification ROC of component 2 versus B cell or CD8 TEM IL6Rα or PD-L1, as well as Treg IL2Rɑ, the CPD component still led to the optimal separation of subject status (Fig. 5h). Thus, in total, the receptor changes we observed represent a coordinated pattern of immunologic reprogramming across cell types and receptors.

### Receptor abundance defines cell type- but not BC-specific responses

**Fig. 6:**
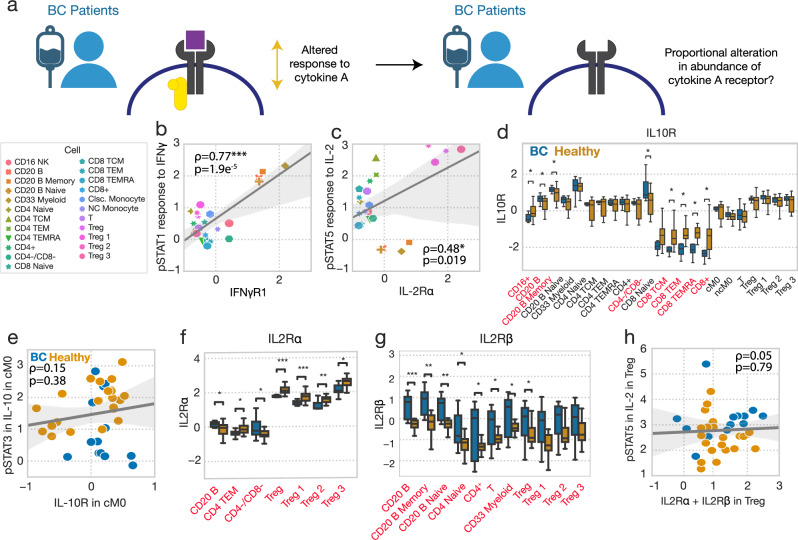
Receptor abundance defines cell type- but not BC-specific responses. **a** Schematic demonstrating our approach to identifying whether subject-level changes in cytokine response can be explained by subject-level alterations in receptor abundance. **b** Average IFN-induced STAT1 phosphorylation versus IFNγR1 abundance across subjects for each cell type (n = 23). Induced responses are reported as z-scored delta MFI. All reported correlations are Pearson correlations. **c** Average IL-2-induced pSTAT5 versus IL2Rɑ abundance across subjects for each cell type. **d** IL10R abundance across cell types, grouped by subject status (healthy n = 22, BC n = 14). Cell types which feature statistically significant responses between BC and healthy status are listed in red. **e** IL-10-induced pSTAT3 versus IL10R abundance in classical monocytes, across subjects (n = 36). **f** IL2Rɑ abundance across cell types, grouped by subject status. **g** IL2Rβ abundance across cell types, grouped by subject status. Significance was derived using the Mann–Whitney U test, comparing those measurements from healthy donors to those of BC patients. Only cell types with significantly altered receptor abundances across cohorts were included in (**f**, **g**). **h** IL-2-induced pSTAT5 versus IL2Rɑ + IL2Rβ in Tregs for each subject. For all box plots, the center line denotes the median, the box limits denote the upper and lower quartiles, and the whiskers denote the 1.5x interquartile range. *, **, and *** represent p values less than 0.05, 0.005, and 0.0005, respectively.

Having measured both cytokine responses and the abundance of these cytokine’s cognate receptors, we next wondered whether receptor-level differences in cytokine signaling regulation could explain the differences in basal and induced signaling in BC patients (Fig. 6a). Receptor expression clearly defined which cell types respond to certain ligands. For instance, average IFNγ-induced STAT1 phosphorylation across cell types correlated with IFNγR1 abundance (Fig. 6b). IL2Rɑ abundance also correlated with which T cell subsets were most responsive to IL-2 (Fig. 6c). Thus, we wondered if subject-to-subject differences in response could also be explained by variation in a cytokine’s cognate receptors.

One of the most prominent changes we observed was a reduced response to IL-10 in BC, and so we first examined whether IL10R was expressed at a lower level in BC. Although in BC patients IL-10 response was reduced across all cell types, we only observed a reduction in IL10R abundance within CD8 cells and B cells across our BC cohort (Fig. 6d). Consequently, while IL-10 responses were significantly reduced in classical monocytes, there was no correlation between blunted responses and the levels of IL10R (Fig. 6e).

Similarly, while IL-2 responses were uniformly increased across cell populations in BC, IL2Rɑ was unchanged in most cell types, and actually decreased in Tregs (Fig. 6f). IL2Rβ was heterogeneously expressed across BC patients, such that it was increased in a subset of patients (Fig. 6g). However, because of this heterogeneity, even the sum of IL-2 receptors did not explain altered IL-2 response in T_reg_s (Fig. 6h). Separate analysis also indicated that neither IL2Rɑ, IL2Rβ, nor their combination in T_reg_s or CD8^+^ cells correlated with IL-2 response across patients.

Differences in receptor amounts also did not have a 1-to-1 correspondence with baseline pathway activation. The general increase in basal pSTAT4 was not explained by IL12RI levels, which were generally found to not significantly vary between healthy donors and BC patients (Fig. S9l). TGFβ-RII was upregulated in CD8^+^ cells, which may partially explain their increased basal pSmad2/3 levels, but not the increases in other cells (Fig. S9b). While the IL6Rα was increased in BC across many populations (Fig. S9h), we did not observe an increase in BC-associated IL-6-induced pSTAT3 response, but rather decreased responses (Fig. S8c). Thus, we determined that the presence of a cytokine’s cognate receptor defined which immune populations respond to a cytokine stimulus (Fig. 6b, c), but not variation in response across subjects (Fig. 6e), including variation which was associated with BC status (Fig. 6h–k).

Finally, to explore the associations between receptor levels and basal / induced signaling in a cell type- and response/receptor-specific manner, we calculated the partial correlation across subjects for each induced or basal phosphorylation level found to be significantly altered between healthy donors and BC patients, as well as each differentially expressed receptor between healthy donors and BC patients in CD4^+^ and CD8^+^ cells. We used hierarchical clustering to examine the correlation patterns and found significant numbers of correlated responses and receptors (Fig. S10a, b). By using partial correlations, we corrected for confounding relationships between our large number of measurements within these populations. We then calculated the correlations between all components across both factorizations of the response and receptor data which were found to vary according to BC status, and again observed a large proportion of statistically significant correlations (Fig. S10c). Taken in sum with our other findings, these results suggest a consistent and coordinated reprogramming of peripheral immunity in cases of BC, where alterations in the regulation, response, and characteristics in one population are accompanied by a litany of other such alterations.

## Discussion

Here, we systematically dissected the altered cytokine signaling responses found in breast cancer patients as compared to healthy controls. Our tensor-based approach to data analysis directly accounted for the multidimensional nature of cytokine response, which varies according to stimulation, cell type, measured signaling product, as well as on a per-subject basis, and critically, according to disease status. CPD allowed us to easily visualize and interpret these multidimensional signatures of response found to be strongly correlated with breast cancer status. These patterns likely would have been obscured using traditional statistical approaches. We also observed patterns of changes in receptor abundance which were coordinated between cell types. However, while we observed that variation in receptor abundance was associated with response to the cognate cytokine across cell types, it did not directly explain the differential responses between subjects. Thus, we found that cytokine response abnormalities likely occur through coordinated upstream programs.

Our work demonstrates the value of two methodological approaches. First, profiling and analyzing cell perturbations such as cytokine responses in a multidimensional manner can reveal coordinated changes across cell types. Specifically, our tensor-based analysis identified various patterns of BC-associated dysregulation in response that included signatures localized to baseline levels of signaling, localized to cell types, or even global changes across cell types. Restricting any one of the dimensions we explored— subjects, cell types, cytokines, or responsive proteins and markers—would have consequently restricted our conclusions, as demonstrated by the limited scope of the findings of our univariate analyses of signaling response and receptor abundances (Fig. S8, S9). Second, including healthy controls provided a baseline for immune response, from which we could contrast the responses observed in cancer samples. Breast cancer, like so many malignancies, demonstrates heterogeneity both within and between patients. However, this work shows that there are consistent and substantial changes across patients, potentially reflecting concerted tumor-directed reprogramming that is critical for disease progression. Understanding these coordinated patterns and their underlying mechanisms may be key to informing the development of more effective integrated immunotherapy-based treatment strategies.

A central finding of our work is that there is a coordinated, widespread, and substantial disruption in the cytokine responsiveness of PBMCs in breast cancer patients, compared to healthy controls, across several pathways, cytokines, and cell types. High basal levels of pSTAT4 and pSmad2/3 were associated with ER^+^ BC and with suppression of STAT3 phosphorylation, at baseline and in response to IL-10. Additionally, this increase in basal pSTAT4 and pSmad2/3 was correlated among BC patients with reductions in IL10R, IL12R, and IL4Rα in B cells and CD8^+^ T cells. This result was particularly interesting as IL-10 is known to drive immunosuppression in most populations, but also more recently been shown to be a key driver of CD8^+^ T cells’ antitumor activity by driving both the tumor infiltration of CD8^+^ cells and their antigen-specific antitumor function upon infiltration^29,30^. Heightened pSmad2/3 through increased TGFβ has been shown to drive cytotoxic cell exhaustion^23^. Likewise, pSTAT4, which has been shown to be associated with increased IL-6 production, was found at significantly elevated levels in unperturbed BC immune cells^31^. We also observed that increased PD-L1 expression was strongly correlated with TGFβ-RII expression, suggesting that these two immunosuppressive proteins work in tandem in CD8^+^ cells to suppress effector response. Indeed, in a recent study profiling peripheral immunity in cancer patients, increased IL-12 signaling activity across populations (presumably through pSTAT4 induction) and exhaustion of CD8^+^ cells accompanied by widespread immune suppression were each found to be associated with cancer status^32^. Interestingly, we also found that IL6Rα expression was negatively correlated with pSTAT3 response to IL-10, indicating that heightened IL6Rα and IL-6 in cancer patients may be dominating STAT3 signaling and in turn suppressing the IL-10 responsiveness. In B cells, in addition to increased basal pSTAT4 and pSmad2/3, we found reductions in IL-10 responsiveness through pSTAT3, increased IFNγ responsiveness through pSTAT1, and increased PD-L1, IL6Rα, and IL2Rβ expression were associated with BC status. Lower pSTAT3 responsiveness to IL-10 potentially reflects potentiation after exposure to the cytokine, and positive staining for PD-L1 is a hallmark of IL-10-secreting regulatory B cells which promote tolerance in allergy and autoimmunity and have previously been associated with invasive breast cancer^33^. Finally, several patterns associated with Tregs were also found to be informative of BC disease status, such as decreased IL2Rα, and increased responsiveness to IL-2 through pSTAT5. These results are intriguing and at first glance contradictory, as IL2Rα potentiates Tregs sensitivity to IL-2^11^. These results may point to Tregs with low activation due to IL-2 starvation, as other B and T cell subsets were found to express high amounts of IL2Rβ (Fig. S9)^34^. Having identified these patterns of dysregulation, it is important to note that while we did have access to patient ages, which were considered during our analysis, we did not have access other patient-matched characteristics, and thus could not consider whether variation arose due to factors such as PR status, which has previously shown to affect cancer immune phenotype^35^.

Taken together, our results point to an overall shift in immunologic features commonly associated with dysregulated immune responses in other contexts. Elevated pSTAT4 levels in T cells is suggestive of a Th17-like response^36^. Indeed, Th17 cells are found in greater numbers within breast tumors, and another inducer of Th17 responses, IL-6, is elevated in various cancers, and plays a central role in the systemic inflammation associated with cachexia^37–39^. A recent mass cytometry-based study of peripheral immunity similarly reported increased Th17-like characteristics in BC patients^40^. Decreases in IL2Rα abundance in the Tregs of breast cancer patients mirrors findings which has been reported in a broad panel of autoimmune diseases^41^. Our analysis also revealed several patient features that correlate with more traditionally cancer-associated immunosuppressive features, which were like those found in the suppression of autoimmune diseases. For example, we found that B cells displaying B_reg_-like phenotypes were selectively found in BC patients. These cells have previously been found to be important to the resolution of autoimmune diseases such as rheumatoid arthritis and experimental autoimmune encephalomyelitis^42,43^. Furthermore, we found that CD8^+^ cells in breast cancer patients exhibited heightened levels of PD-L1 and reduced capacity to respond to IL-10. These features are typical of regulatory CD8^+^s, a cell type previously reported as resident in breast cancer tumors and with important roles in the control of systemic lupus erythamatosis^44,45^. Thus, the patterns of signaling unique to breast cancer patients are reminiscent of systems where inflammatory features are only held in check by compensatory increases in several immunosuppressive cell types and signaling processes, a state which resembles that of suppressed autoimmune responses.

An important distinction of our work is that, while most characterization of tumor immunity has focused on the local microenvironment or lymph nodes, our observations were made in peripheral cells with no selection for antigen-specific cells^46^. Furthermore, we find that several changes in cytokine responsiveness and marker expression previously associated with breast cancer are, in fact, coordinated changes, which could be the result of a shared process of tumor-driven reprogramming^47^. Previous work has shown that these global changes are not explained by changes in cytokine abundance in the blood^6^, and thus may be a result of immunologic reprogramming within lymph nodes^48^, or reprogramming during local trafficking of cells through the tumor.

While we were able to establish differences in cytokine response and baseline receptor abundance, these data establish a strong basis for a more in-depth characterization of the global immunologic differences that develop with cancer. We consistently found that our results were mirrored in similar studies of dysregulation of peripheral immunity, suggesting that the programs of dysregulation identified in this study, despite limitations in our cohort size, are representative of consistent patterns of immune alterations in other cancer contexts^2,32,40^. Cancers develop through a progressive process of immunoediting and then escaping from immune control^49^. Analyzing the changes in cytokine responsiveness and receptor abundance we identified here, alongside other forms of profiling information such as transcriptional and epigenomic, will help to reveal both how these cytokine pathway changes arise and how they are linked to functional changes within immune populations^50,51^.

An open question is also how these changes within the periphery reflect changes within other immune sites, such as lymph nodes and the tumor microenvironment^7,46^. One might expect that through circulation peripheral cells reflect changes in the local tumor microenvironment^7^, or that differential effects in trafficking mean that the periphery reflects the absence of certain cytokine-responsive cells, or presence of cells which stymie response^52^. While our previous work has shown that dysregulated signaling response correlates with relapse^2^, profiling these coordinated changes in other patient populations that vary in outcomes and treatment response, along with identifying other features correlated with cytokine responsiveness, may allow us to more fully assess how these changes might provide a useful prognostic readout of immune functionality.

## Methods

### Human samples

Peripheral blood samples were obtained from consented patients (IRB #21368 and #19186) with ER^+^ breast cancer at City of Hope. Patient characteristics are summarized in Supplemental Table S1. All patients who consented to this study had no previous history of breast cancer and were estrogen receptor-positive (ER^+^ ), HER2/neu negative (HER2^-^). Patient’s blood was drawn into EDTA-containing tubes. Peripheral blood mononuclear cells were isolated by Ficoll-Paque (Cytiva, Marlborough, MA, USA) density centrifugation following the manufacturer’s protocol and cryopreserved in 10% DMSO FBS. Age-matched healthy control peripheral blood samples were obtained from City of Hope Blood Donor Center.

### Cell culture

Cryopreserved PBMCs were thawed and rested overnight (16 h) in RPMI 1640 medium supplemented with 10% fetal bovine serum, 1% penicillin-streptomycin-glutamate (PSG) at 37°C, 5% CO_2_. PBMCs were counted using a hemocytometer, and viability was determined by trypan blue exclusion (Sigma-Aldrich). Subsequently, cells were stimulated in 96 deep-well V plates at a concentration of 0.5–1 × 10^6^ cells/ml in fresh RPMI 1640 medium (Thermo Fisher Scientific Inc., MA) supplemented.

### Cell signaling

After resting period, PBMCs were either untreated or stimulated with IFNγ (50 ng/ml), IL-10 (50 ng/ml), IL-6 (50 ng/ml), IL-4 (50 ng/ml), or TGFβ (50 ng/ml) (Peprotech, Rocky Hills, NJ, USA) at 37°C for 15 min, followed by fixation with 1.5% paraformaldehyde (PFA) for 10 min at room temperature. Following PFA fixation, cells were washed with PBS 1x and permeabilized using ice-cold 100% methanol. Following methanol fixation, cells were stored at –80°C. Cells were then washed three times with staining buffer (PBS supplemented with 1% FBS) before antibody staining.

### Phospho flow cytometry

The following antibodies were used: STAT4-AF647 (clone 38/p-Stat4), CD14-APC-Cy7 (clone HCD14), CD20-AF700 (clone H1), STAT6-V450 (clone 18/pStat6), PD-L1-BV510 (clone 29E.2A3), CD3-BV570 (clone UCHT1), PD1-BV605 (clone EH12.1), CD33-BV750 (clone p67.6), CD27-BV786 (clone L128), CD45RA-BUV395 (clone HI100), CD4-BUV563 (clone SK3), CD16-BUV737 (clone 3G8), CD8-BUV805 (clone SK1), STAT3-AF488 (clone 4/p-Stat3), STAT1-Percp-Cy5.5 (clone 4a), SMAD2/3-PE (clone O72-670), Foxp3-PE-CF594 (clone 259D/C7), STAT5-PE-Cy7(clone 47). Dilutions of antibodies were prepared based on the manufacturer’s recommendations and optimized for staining conditions in preliminary experiments. Incubation was carried out for 45 min at room temperature. All antibodies used were purchased from Biolegend San Diego, CA, USA or BD Biosciences (Franklin Lakes, NJ, USA).

### Cytokine receptor flow cytometry

The following antibodies were used: CD45RA-BUV395 (clone HI100), CD3-APC-Cy7 (clone UCHT1), CD16-BUV737 (3G8), CD16-APC-Fire810 (3G8) CD119 (IFNγR1)-BB660 (clone GIR-208), CD33-BV750 (clone p67.6), CD27-AF700 (clone O323), CD14-BUV496 (clone MOP9), CD132 (IL-2Rγ)-BB700 (clone TUGh4), CD210 (IL-10R)-PE-Cy7 (clone 3F9), CD122 (IL-2Rβ)-BV786 (clone MIKB3), TGF-βR2-APC (clone W17055E), PD-L1-BV510 (clone 29E.2A3), CD212 (IL-12R1)-BV421 (clone 2.4e6), PD1-BV605 (clone EH12.1), CD8-BUV805 (clone SK1), CD4-BUV563 (clone SK3), CD25-BB515 (clone MA251), CD124 (IL-4Rα)-BB630 (clone HIL4R-M57), CD124 (IL-4Rα)-PE (clone HIL4R-M57), CD130 (IL-6Rβ)-PE-CF594 (clone M5), CD130 (IL-6Rβ)-BUV737 (clone M5), CD127-BV650 (clone A019B7). Dilutions of antibodies were prepared based on the manufacturer’s recommendations and optimized for staining conditions in preliminary experiments. Incubation was carried out for 30 min at 4^o^C. All antibodies used were purchased from Biolegend San Diego, CA, USA or BD Biosciences (Franklin Lakes, NJ, USA).

### Data acquisition and gating strategy

Stained cells were analyzed using the Cytek Aurora flow cytometer with a 355 nm, 405 nm, 488 nm, 561 nm, and 640 nm laser configuration. Compensations were established using single-stained controls and a negative control sample. A total of 50,000 to 100,000 events were acquired at a data rate of 1000 events/s. Cell populations were gated as shown in Supplementary Figs. S1. Experiments were conducted once for each measurement.

### Gating and preprocessing

For all quantification of cellular species abundances, whether cell type markers, signaling products, or receptor amounts, the mean fluorescent intensity (MFI) of flow cytometry data was calculated to determine signal. Cell population gating was performed as shown in Fig. S1. Before decomposition, the mean signaling response data was background subtracted on a per-subject, per-cell type basis, and normalized for each signaling marker according to the maximum signal observed for that marker. Receptor data was normalized by z-scoring each receptor’s signal.

### Canonical polyadic decomposition

Before analysis, we reorganized our measurements into a third- or fourth-order tensor, i.e., a three- or four-dimensional array with axes representing parameters over which the profiling was conducted. Within the CP decomposition model, a tensor is described as a sum of the tensor product ($\otimes$) of rank-one components that represent the contribution of each mode. For instance, in the three-mode case:

1 $$X=\mathop{\sum }\limits_{r=1}^{R}{a}_{r}\otimes {b}_{r}\otimes {c}_{r}$$

where ${a}_{r}$, ${b}_{r}$, and ${c}_{r}$ are the r^th^ column of the factor matrices $A$, $B$, and $C$, which overall summarize how each pattern is represented across the three dimensions.

While many algorithms exist for deriving CP factorizations, we applied alternating least squares (ALS), wherein each mode is iteratively solved using least squares. As an iterative procedure, ALS must be initialized with a starting estimate of the factorization. The CPD decomposition was initialized using the right-hand $r$ eigenvectors from SVD decomposition of the tensor flattened along each mode.

ALS applies the observation that, given factor matrices for two of the modes (e.g., modes 2 and 3), the optimal factor matrix for the remaining mode can be solved for as the least squares solution between the tensor unfolding and Khatri-Rao product of the known factors:

2 $$\mathop{\min }\limits_{A}{\Vert {X}_{(1)}-A[{(C\odot B)}^{T}]\Vert }^{2}$$

*X*_(1)_ represents the tensor unfolding of *X* along mode 1, and *C*⊙*B* represents the Khatri-Rao product of $C$ and $B$. After solving for $A$, ALS proceeds for each of the remaining modes, completing one iteration of the algorithm by building a representation of the other factors using the Khatri-Rao product, and then applying least squares to solve for the left-out factor matrix.

In addition to the ALS scheme described above, we applied the line search routine described by Bro^53^. Briefly, after two rounds of ALS, the difference between the last two fitting iterations was used to look ahead by a line search step equal to $\root{l\,}\of{N}$, where $N$ is the iteration number and $l$ is the line search quotient, initially equal to 2. If the error of the line search-updated factorization is less than the normal ALS update, then the line search result was accepted. Otherwise, the ALS result was used. After four straight rejections the line search quotient was increased by 1 to reduce the line search distance.

### Censored alternating least squares

The censored least squares algorithm is solved similarly to ALS but differs in its approach of handling the missing value problem. Rather than imputing missing values, we first grouped columns during the least-squares solve based on their pattern of missing values within the dataset. Solving was then performed on each group, with the missing rows removed during each part of the solving process. In this way, the least squares solution was solved without inclusion of the missing values or the need for imputation during fitting.

### Tucker decomposition

Tucker decomposition was performed using TensorLy’s implementation of Tucker decomposition using randomized SVD initialization^54^. Rank search was conducted by performing increasing the rank of each mode iteratively, following the Pareto front of the smallest factorization to produce the lowest reconstruction error.

### Logistic regression

Regularized logistic regression was implemented using the implementation provided by scikit-learn, with an l1 penalty^55^. Solving was performed with the Stochastic Average Gradient (SAGA) solver^56^, a maximum iteration number of 5,000 and tolerance of $1.0\times {10}^{-6}$. The regularization strength was determined through cross-validation using the LogisticRegressionCV and the default parameters regarding attempted regularization strengths. We applied repeated, stratified, 10-fold cross-validation, with 20 repeats throughout the analysis.

### Factor match score

The factor match score (FMS) is a quantification of the consistency of CPD factors^57^. We utilized the FMS to ascertain the stability of our CPD results by comparing our factorization of our signaling and receptor datasets to subsets of the data generated by jackknife sample of these datasets across patients. We then calculated the FMS of the factors generated via factorization of our original complete dataset, to each of the 36 factorizations of our jackknife data subsets FMS was calculated across the cytokines, time, and cell type factors of our signaling dataset, and the receptors and cell type factors of our receptor dataset, as jackknife sampling resulted in factorizations with distinct patient mode sizes. To compensate for potentially divergent component orders, a linear sum assignment is also applied during the FMS calculation. An FMS of 1 is indicative of perfectly consistent factors.

3 $${FMS}=\mathop{\sum }\limits_{r=1}^{R}\left(1-\frac{{w}_{i}{w}_{i}}{\max \left({{w}_{i}w}_{i}\right)}\right)\,\cdot\, \frac{{A}_{i}^{T}{A}_{j}}{\big|{|}{A}_{i}{|}\big\Vert{A}_{j}\big|{|}}\,\cdot\, \frac{{B}_{i}^{T}{B}_{j}}{\big|{|}{B}_{i}{|}\big\Vert{B}_{j}\big|{|}}$$

$$w=\big|{|}A{|}\big|\,\cdot\, \big|{|}B{|}\big|$$

### Quantification and statistical analysis

Descriptions of pertinent statistical analyses or metrics used, the number of replicates of experiments performed, and the values of confidence intervals can be found in each figure caption. n indicates the number of times a particular experiment was performed (duplicate, triplicate, etc.) within each figure.

To test for statistical differences between induced and basal signaling activation, as well as receptor abundance, a Mann–Whitney U was performed, where each point was representative of a single subject’s measurement for that cell type.

The statistical significance of each correlation was determined by permutation test.

## Supplementary information

Supplemental Information

## Data Availability

All experimental data can be found at https://github.com/meyer-lab/tfac-CoH. All other data needed to evaluate the conclusions in the paper are present in the paper or the Supplementary Materials.

## Code availability

All analysis was implemented in Python v3.11 and can be found at https://github.com/meyer-lab/tfac-CoH, release 1.0.
