# Supplementary material for: Systems profiling reveals recurrently dysregulated cytokine signaling responses in ER+ breast cancer patients’ blood
Source: NPJ Syst Biol Appl. 2024 Oct 10;10:118. doi: 10.1038/s41540-024-00447-0 (PMC11467214; doi:10.1038/s41540-024-00447-0)
Supplement: Supplementary file 1 — Supplemental Information [file 41540_2024_447_MOESM1_ESM.pdf]

Supplement

Supplementary Table 1. Breast cancer patient characteristics and demographics.

Age	ER	PR	Her2	Ki67 (%)	TMR Grade	T	N	Stage	Note
48	>95	40	-	1	2	1b	0	Ia	
71	95	60	-	5-10	1	1B	1a	IIa	
55	90	50	-	10	2	1c	1(m)	Ib	
48	90	80	-	10	2	2	0	IIb	Chest wall recurrence 5/2021 BRCA 1/2 -
73	100	90	-	5	1	3	0	IIb	
41	90	40	-	25	2	1b	0	Ia	
42	> 90	70	-	10	1	1b	0	Ia	
51	90	90	-	25	2	2	1a	IIb	
35	>90	80	-	20-30	2	U	U	U	
65	>95	>90	-	10-15	2	T2	0	IIa	

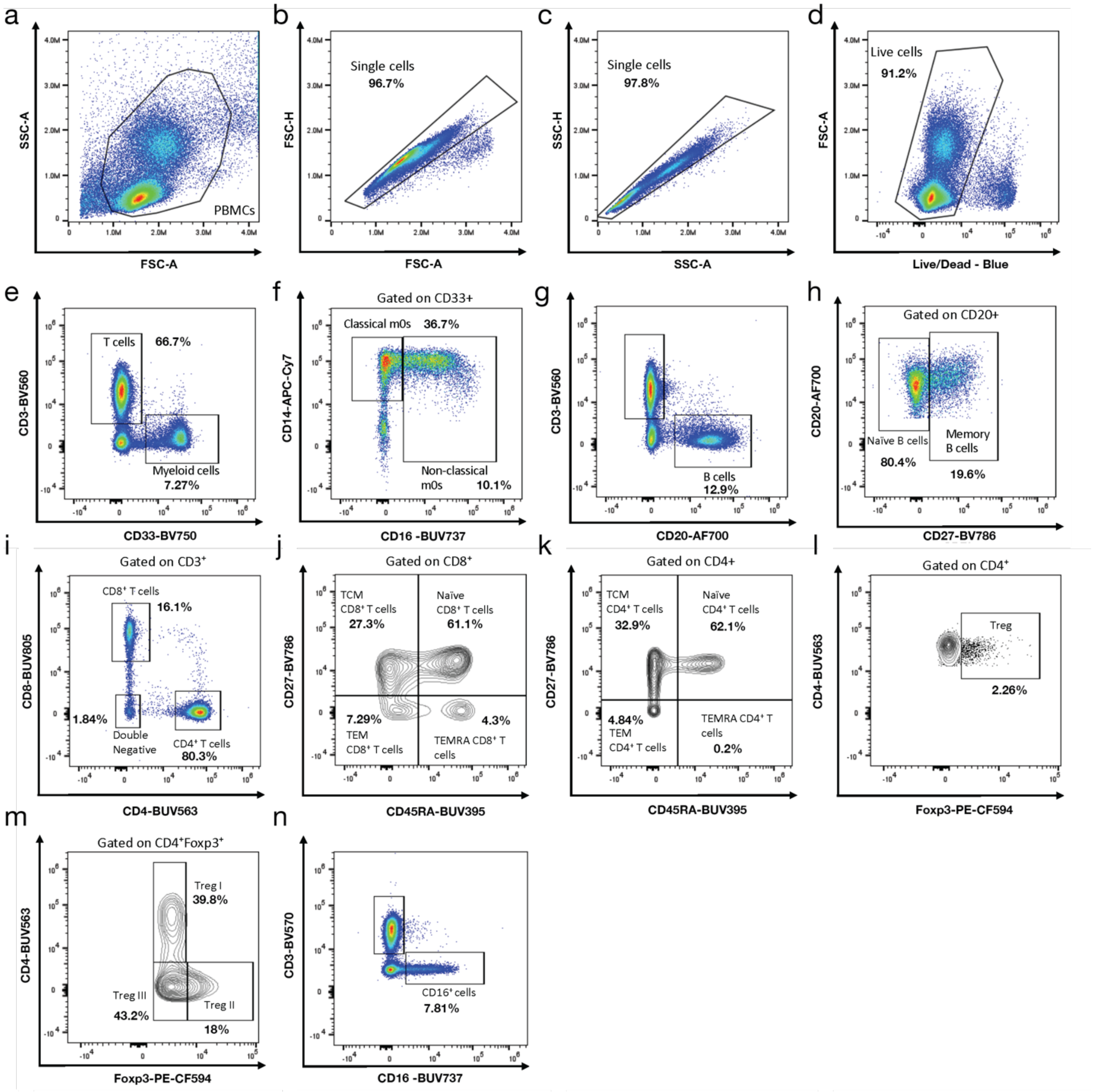

Supplementary Figure 1. Gating strategy for the signaling response and receptor quantification data. (a–d) Gating for live, single cells. **(e)** Myeloid and T cell gating. **(f)** Gating of classical and non-classical monocytes. **(g)** Gating of B cells. **(h)** Gating of memory and naïve B cells. **(i)** Gating of CD8$^+$, CD4$^+$, and CD4$^-$CD8$^-$ cells. **(j)** Gating of TCM, naïve, TEM, and TEMRA CD8$^+$ cells. **(k)** Gating of TCM, naïve, TEM, and TEMRA CD4$^+$ cells. **(l)** Gating of Treg cells. **(m)** Gating of Treg 1, Treg 2, and Treg 3 populations. **(n)** Gating of CD16$^+$ population.

a

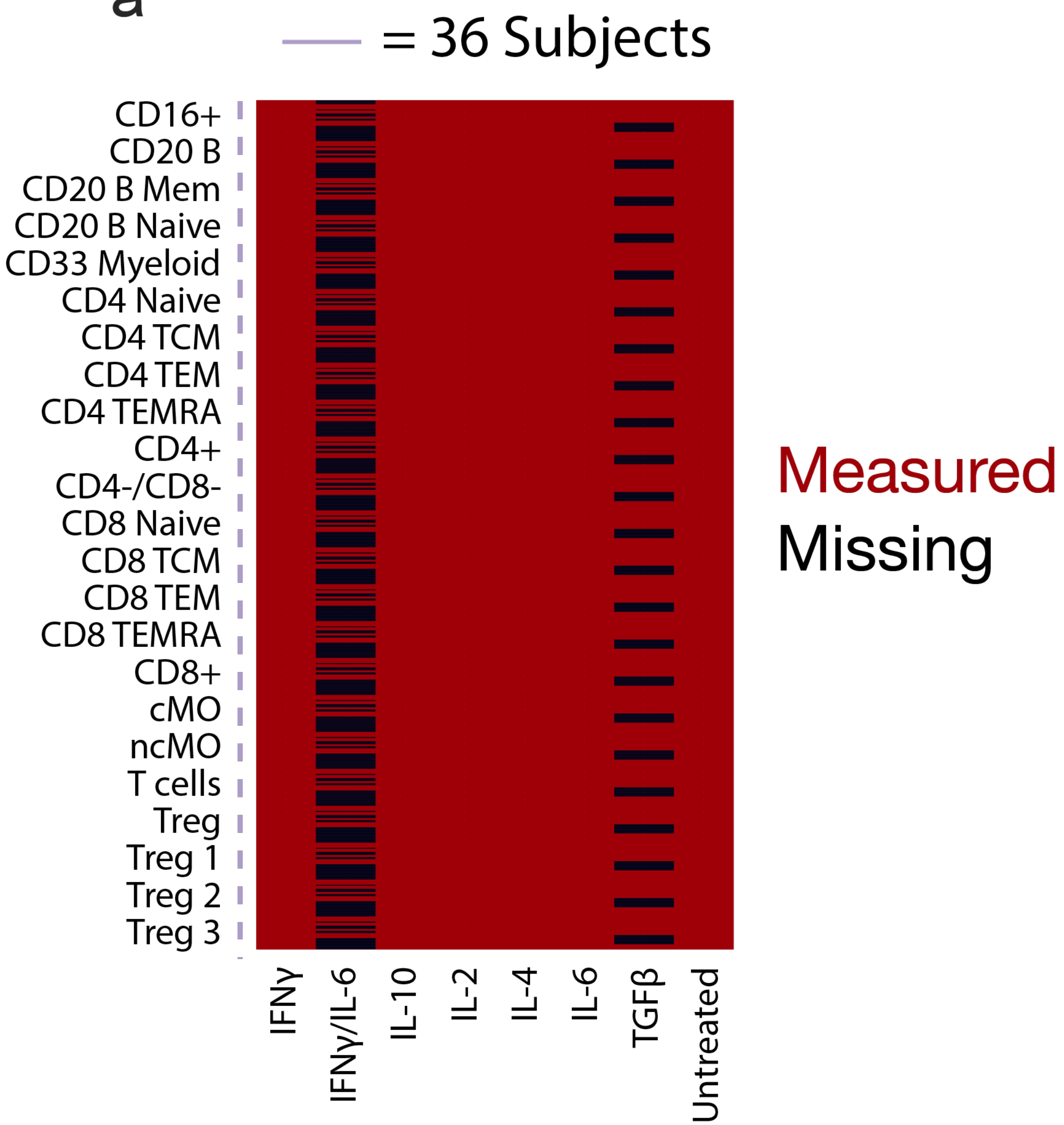

Supplementary Figure 2. Visualization of missing values across cytokine stimulations. (a) Values which were measured across all signaling markers are shown in red. Missing measurements across all signaling markers are shown in black. Missing values are concentrated among cancer patients, whose samples included fewer values.

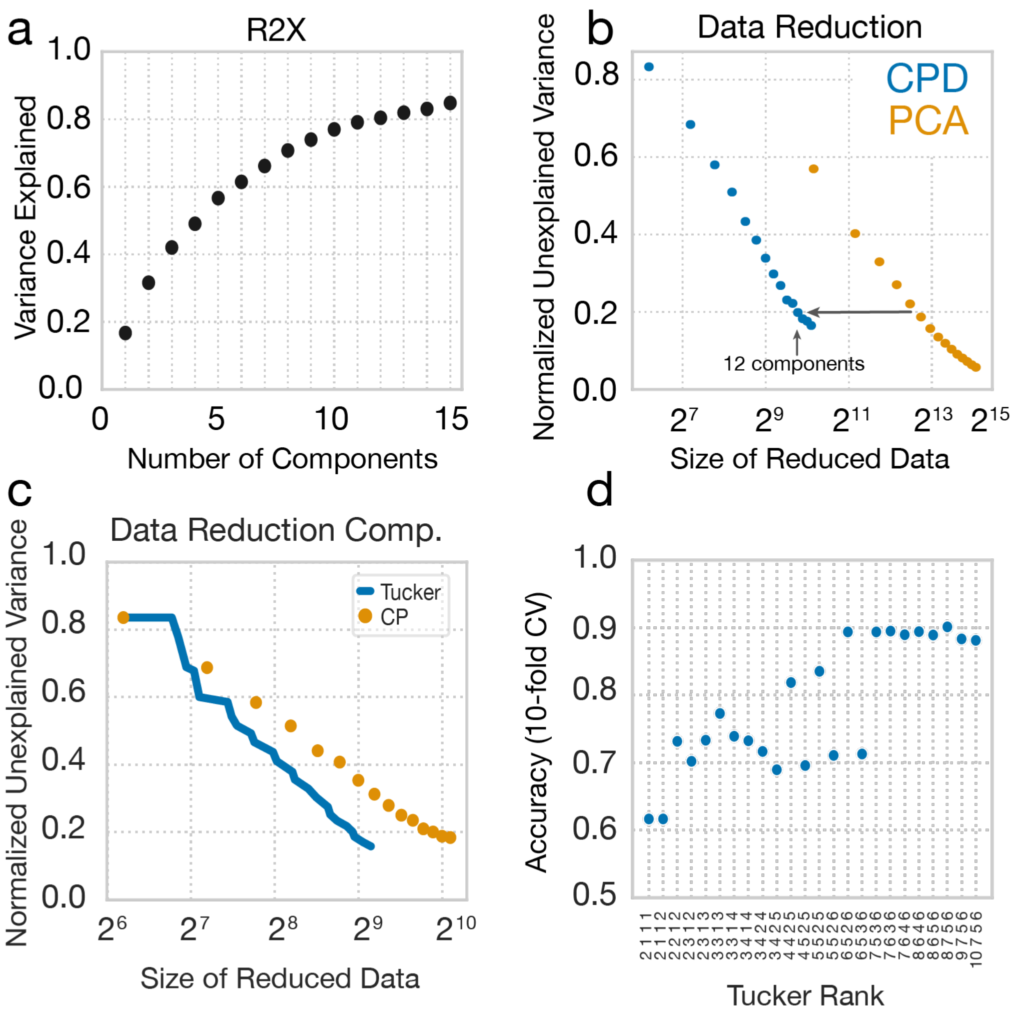

Supplementary Figure 3. CPD efficiently summarizes cytokine response dataset. **(a)** Percent variance reconstructed (R2X) versus the number of components used in CPD. **(b)** The remaining error on reconstruction, normalized to the total dataset variance, versus the size of the dataset after decomposition using CPD or PCA. **(c)** Comparison of percent variance reconstructed for CPD and Tucker decomposition at various data sizes. **(d)** The accuracy of a logistic regression classifier upon 10-fold cross-validation, using the Tucker subject factors with varying numbers of components. Tucker ranks are listed in the X axis; the ranks are listed in order of subject, cytokine, cell population, and signaling marker.

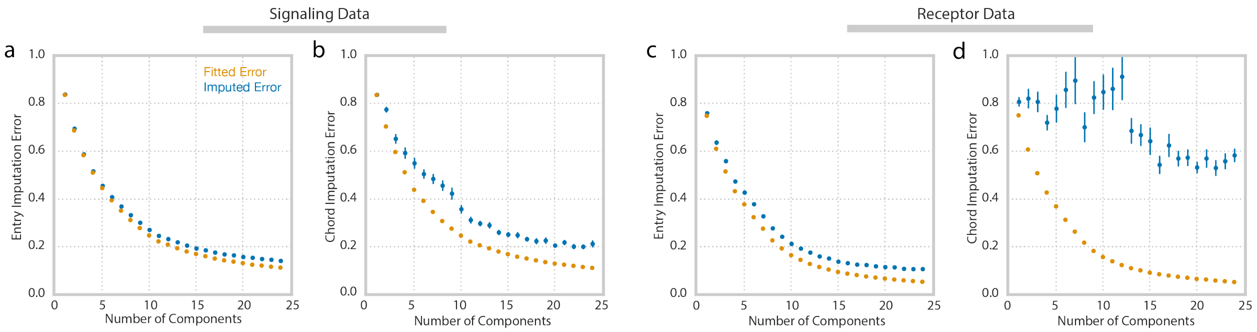

Supplementary Figure 4. CPD can accurately impute the signaling and receptor data. (a,b) The fitting (gold) and imputation (blue) error using decompositions of varying component numbers for cytokine response dataset. Predictions were made by withholding and subsequently imputing (a) 10% of the entries or (b) 10% of the chords along the subject mode. Fitting error was calculated as the variance explained of those same chords when not withheld from dataset. **(c, d)** The fitting (gold) and imputation (blue) error using decompositions of varying component numbers for the receptor dataset. Predictions were made by withholding and subsequently imputing (c) 10% of entries or (d) 10% of the chords along the subject mode.

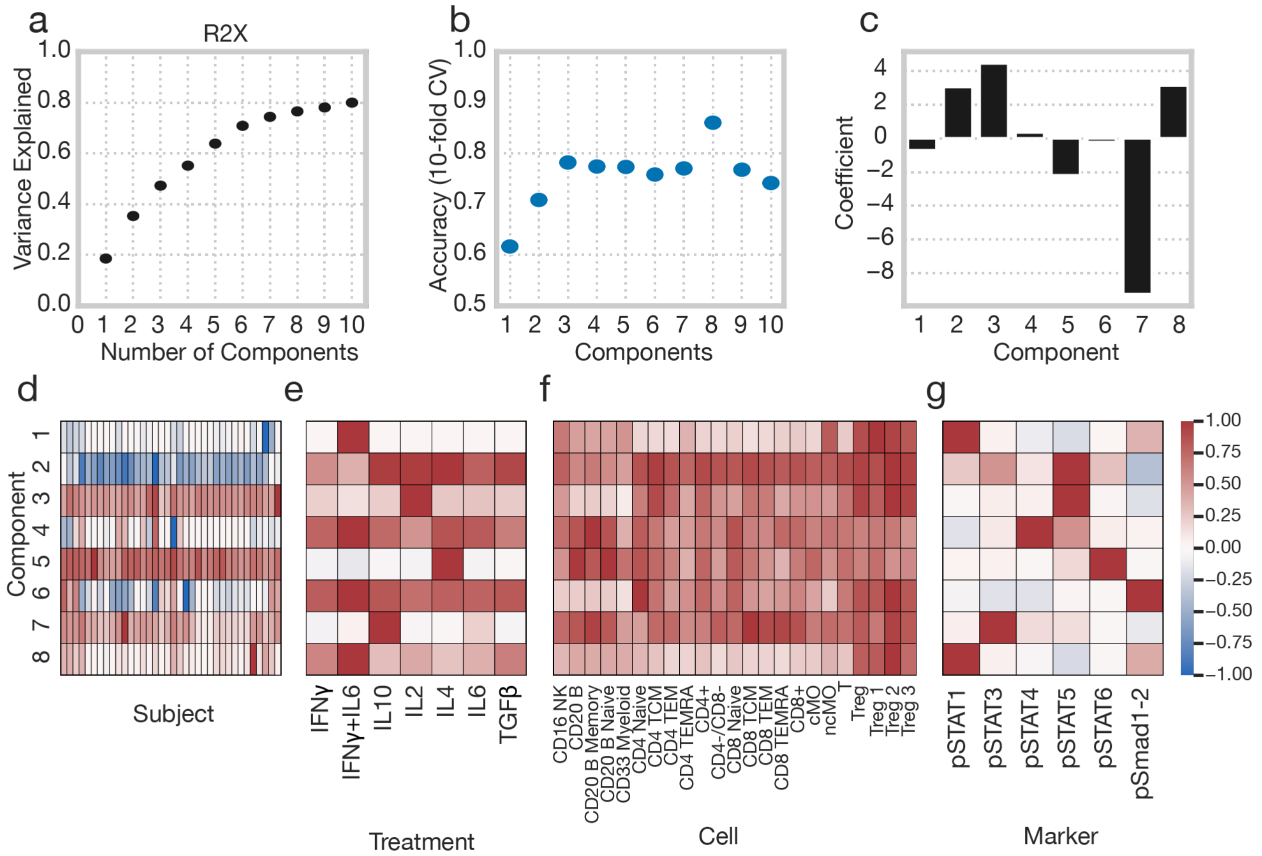

Supplementary Figure 5. Tensor factorization of response data using fold-change metrics reveals similar patterns of dysregulation. (a) Percent variance reconstructed (R2X) versus the number of components used for decomposition of fold-change signaling dataset. Here, the signaling responses were calculated and included as the fold change with respect to untreated controls. Untreated controls are thus not included in the factorization. **(b)** Accuracy of logistic regression disease status classification model fit to subject factors for tensor decompositions of varying sizes. Accuracy was determined via 10-fold stratified cross validation. **(c)** Weights of each component for a logistic regression model fit to subject factors using an 8-component decomposition (Healthy = 0, BC = 1). **(d–g)** Component values for each subject (d), treatment (e), cell type (f), and signaling marker (g) collected using a tensor decomposition with 8 components.

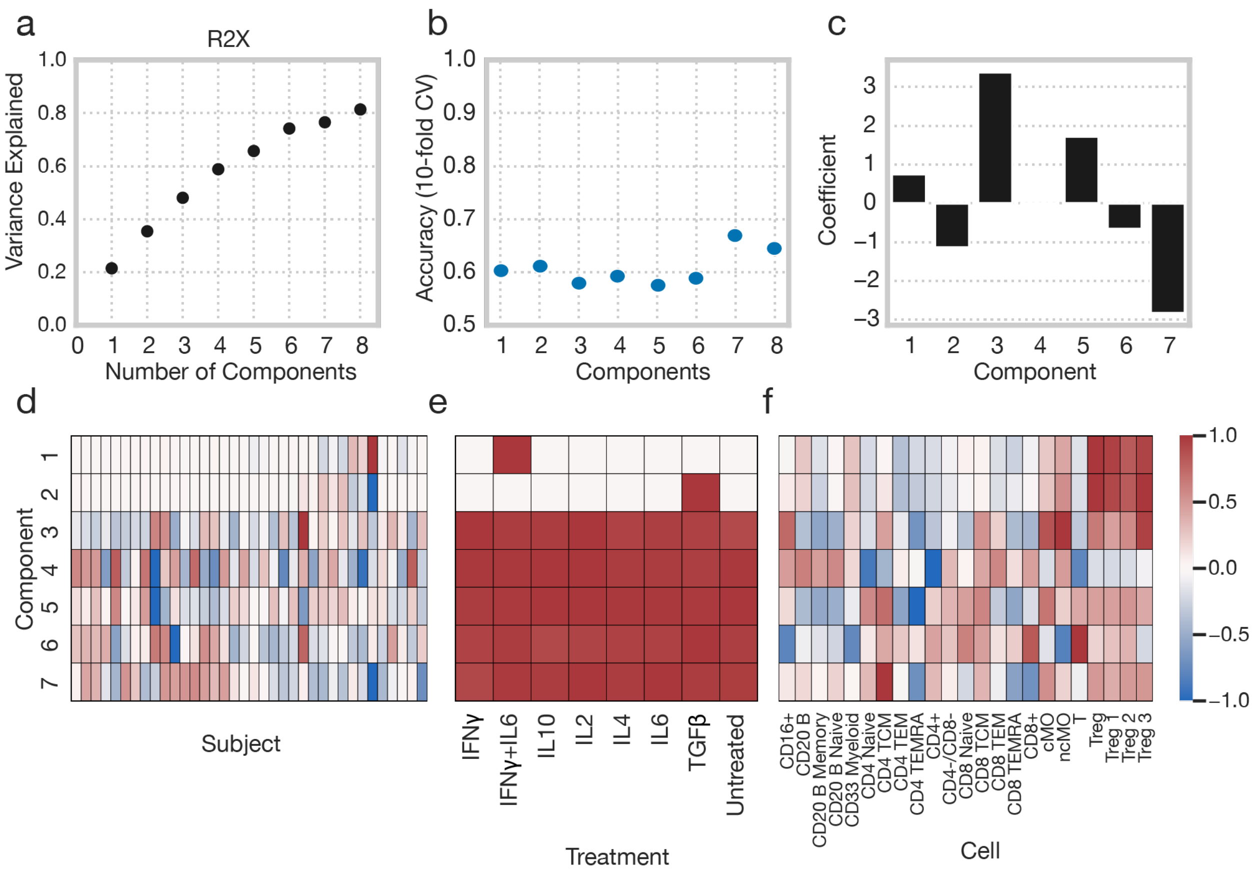

Supplementary Figure 6. Tensor factorization of cell type abundance fails to uncover patterns associated with disease. (a) Percent variance reconstructed (R2X) versus the number of components used for decomposition of cell type abundance dataset. The abundance of each cell type was calculated by using the percentage of total live lymphocytes which they accounted for. Each abundance was calculated on a per-measurement basis (one subject, one cytokine). **(b)** Accuracy of logistic regression disease status classification model fit to subject factors for tensor decompositions of varying sizes. Accuracy was determined via 10-fold stratified cross validation. **(c)** Weights of each component for a logistic regression model fit to subject factors using a 7-component decomposition (Healthy = 0, BC = 1). **(d-g)** Component values for each subject (d), treatment (e), and cell type (f) collected using a tensor decomposition with 7 components.

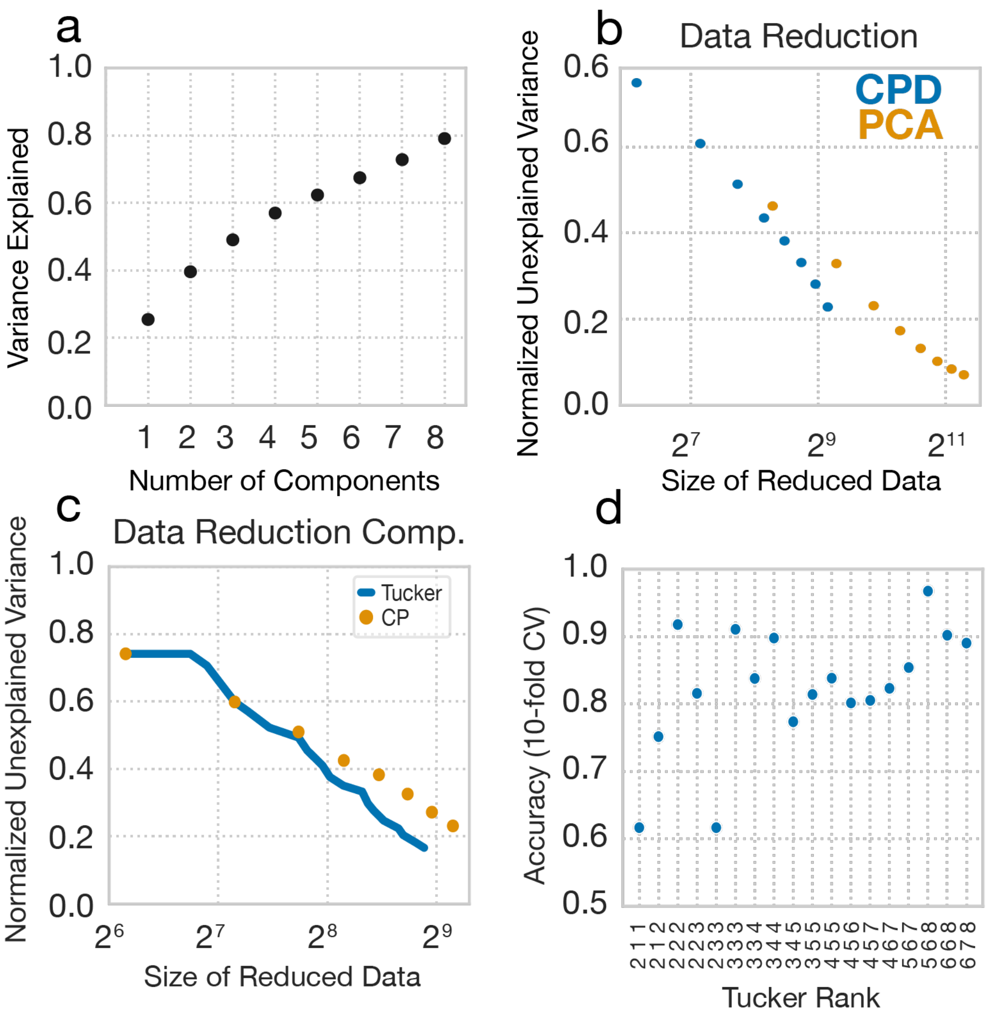

Supplementary Figure 7. CPD efficiently summarizes receptor abundance dataset. **(a)** Percent variance reconstructed (R2X) versus the number of components used in CPD. **(b)** The remaining error on reconstruction, normalized to the total dataset variance, versus the size of the dataset after decomposition using CPD or PCA. **(c)** Comparison of percent variance reconstructed for CPD and Tucker decomposition at various data sizes. **(d)** The accuracy of a logistic regression classifier upon 10-fold cross-validation, using the Tucker subject factors with varying numbers of components. Tucker ranks are listed in the X axis; the ranks are listed in order of subject, cell population, and receptor.

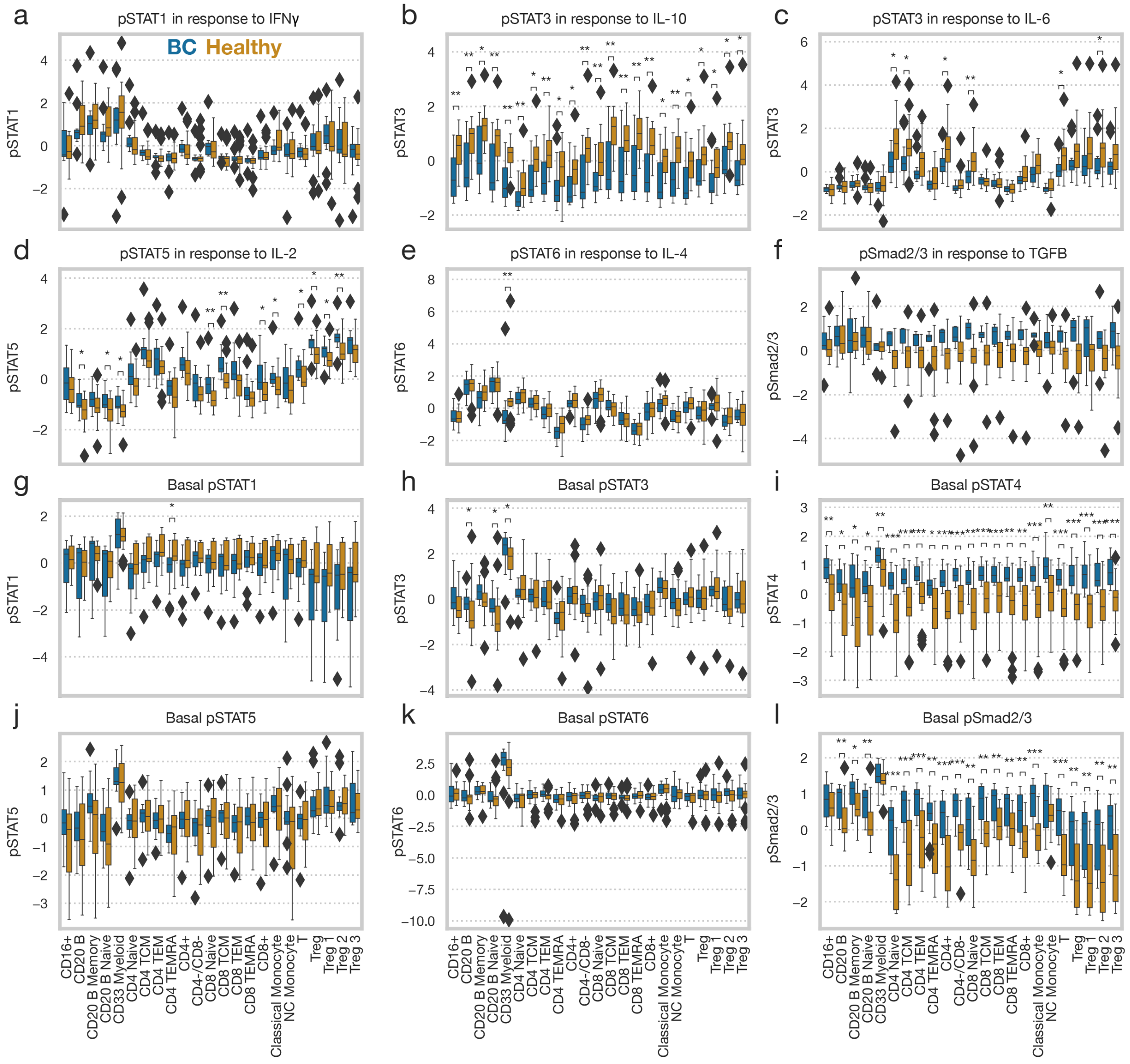

Supplementary Figure 8. Partial panel of signaling responses. Data was stratified according to disease status (healthy n=22, BC n=14). The data was mean centered across all cell types and subjects. Statistical significance was determined by two-tailed Mann-Whitney U test (two-tailed) comparing those measurements from healthy donors to those of BC patients. All basal levels are reported as MFI, and induced responses are measured as ΔMFI. For all box plots, the center line denotes the median, the box limits denote the upper and lower quartiles, and the whiskers denote the 1.5x interquartile range. *, **, and *** indicate a p-value of less than 0.05, 0.005, or 0.0005, respectively.

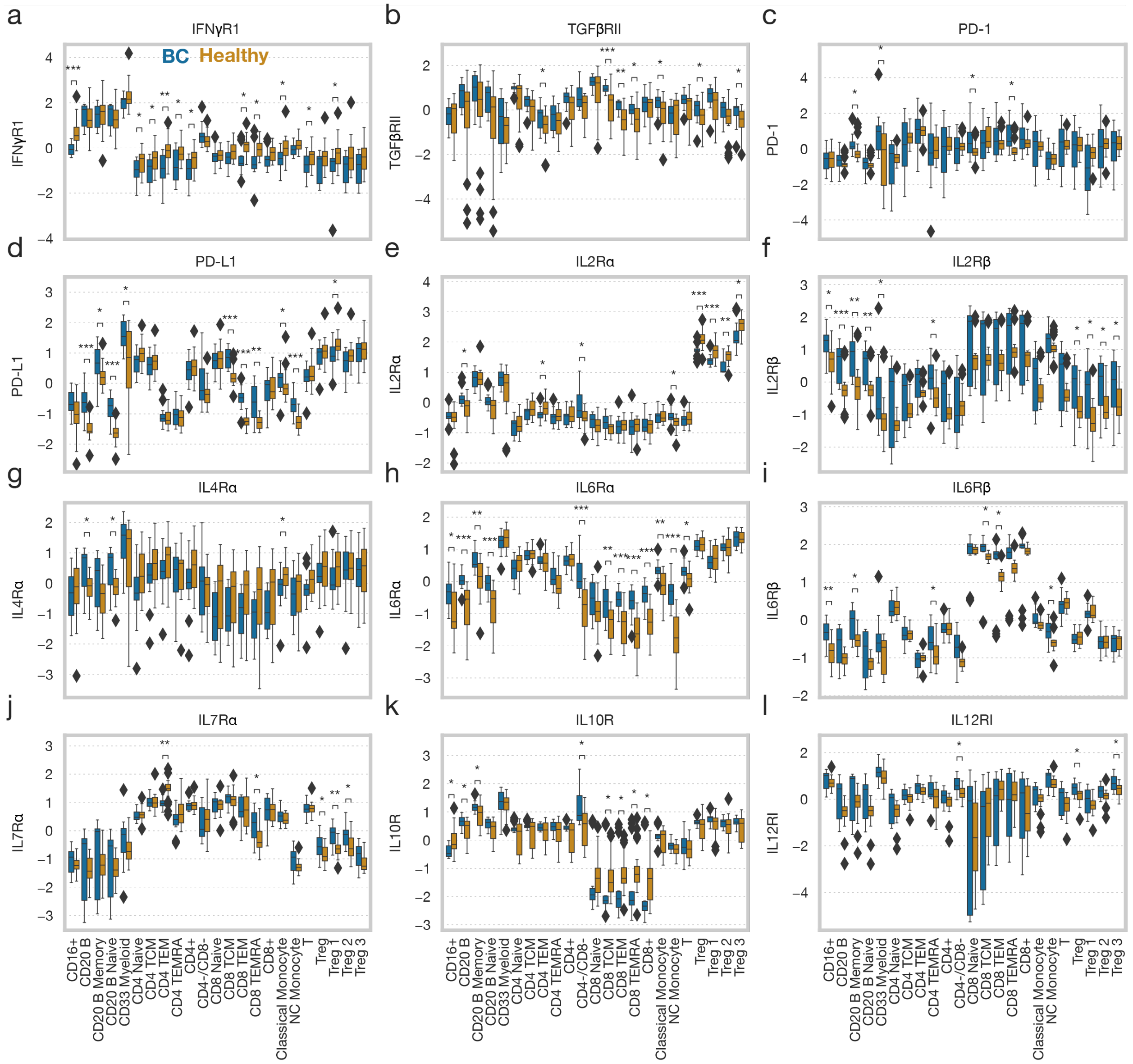

Supplementary Figure 9. The full panel of receptor profiling. Data was stratified according to disease status (healthy n=22, BC n=14). The data was mean centered across all cell types and subjects. Significance was derived using the Mann-Whitney U test (two-tailed) comparing those measurements from healthy donors to those of BC patients. For all box plots, the center line denotes the median, the box limits denote the upper and lower quartiles, and the whiskers denote the 1.5x interquartile range. *, **, and *** indicate a p-value of less than 0.05, 0.005, or 0.0005, respectively.

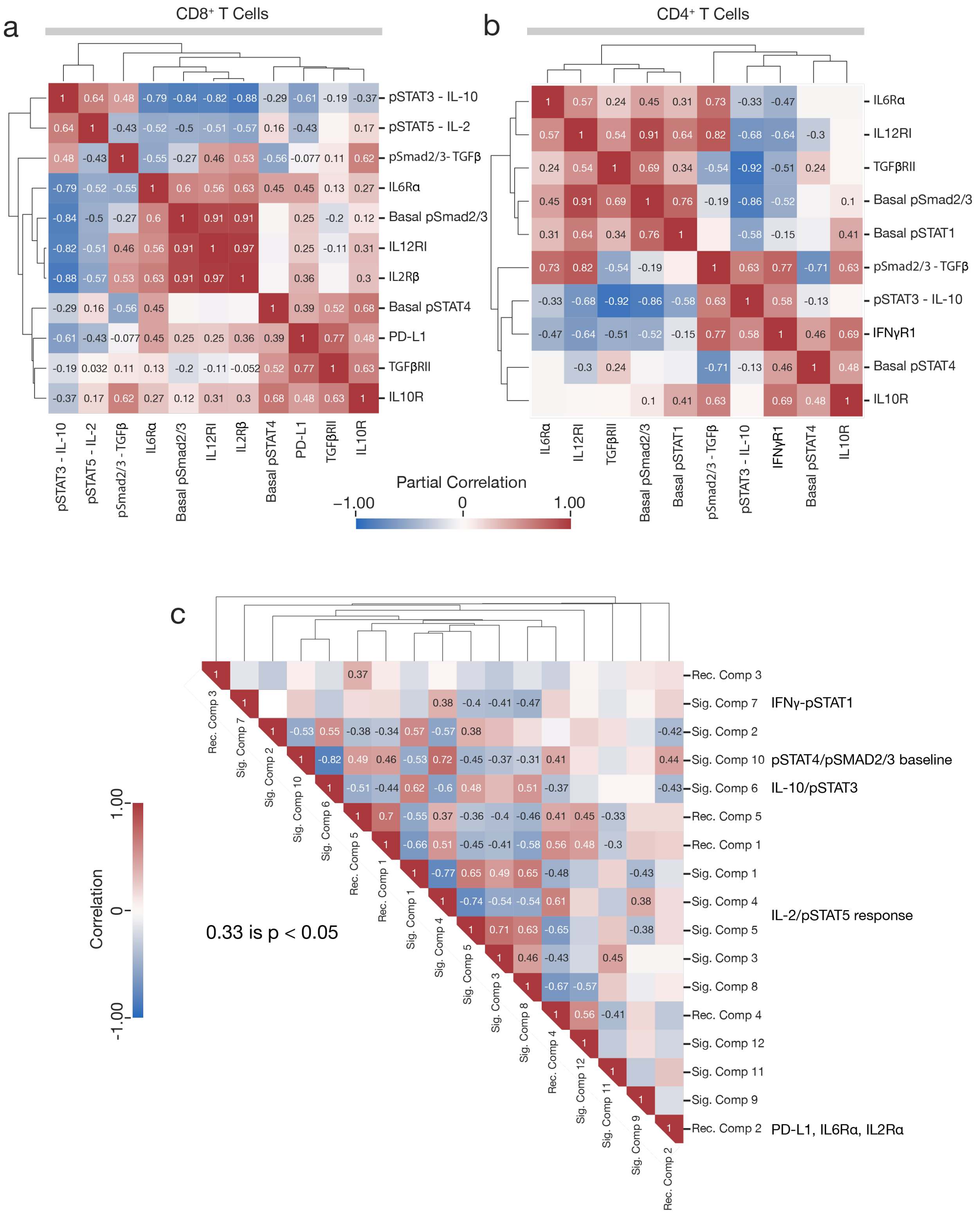

Supplementary Figure 10. Patterns of coordinated receptor-signaling dysregulation suggest mechanisms of response reprogramming both globally and in a cell type-specific manner. (a–b) Partial correlations among measurements with statistically significant differences in CD8⁺ (a) or CD4⁺ (b) T cells. Pearson

correlations were calculated across subjects, specifically within the BC cohort (n=14). Measurements included were those found to be significantly different between healthy donors and BC patients in univariate statistical tests. **(c)** Correlations across all subjects among the receptor and signaling component patterns (n=36). Summaries of the patterns encoded by those components found to be informative of BC status are summarized on the X axis. Statistically significant correlation cutoff was calculated by permutation tests.
